# Vitality structures in ‘addictive’ game design

**DOI:** 10.12688/openreseurope.17177.1

**Published:** 2024-03-01

**Authors:** Veli-Matti Karhulahti

**Affiliations:** 1 Department of Music, Art and Culture Studies, University of Jyväskylä, Jyväskylä, Finland

**Keywords:** behavioural addiction, gambling, gaming, phenomenology, psychiatry, qualitative, technology, theory

## Abstract

For decades, research on technology use and mental health has been based on the assumption that identifiable structures of ‘design’ are psychologically relevant for their users. This has been central especially for the nosological emergence of ‘behavioural addictions’, which currently involve two formal diagnoses involving technology: problems related to playing gambling games (gambling disorder) and videogames (gaming disorder). Alas, the research on identifying ‘addictive’ design structures has suffered from major construct validity issues. To make progress across those issues, I draw from the history of ‘vitality affects’ in psychiatry and introduce *vitality structures* as a design-phenomenological framework that can help researchers conceptualise clinically (and non-clinically) relevant ‘bonds’ between entities of game design and corresponding player phenomenology. Vitality structures are not natural kinds to be discovered but pragmatic constructs to be created and used: they are useful as long as they communicate what is both identifiable and empirically prevalent. As a demonstration of practice, I propose working conceptualizations of three vitality structures, which surface in videogames that have been prevalent sources of self-identified problems among gaming treatment-seekers. Systematic programs of research for identifying relevant vitality structures across technological, psychological, and psychiatric contexts can lead to construct-valid and replicable design effects.

## Introduction

My goal here is to contribute to the interdisciplinary research efforts on what the current psychiatric discourse refers to as ‘addictive behaviours’. I focus on one such behaviour class—gaming, i.e. videogame play—due to its status as one of the two diagnosable mental disorders in the *International Classification of Diseases* (ICD-11) in this category. To better conceptualize those ‘addictive’ videogame mechanisms that the related clinical research programs imply, I propose a dual design-phenomenological framework based on *vitality structures*: construct ‘bonds’ between identified units of experience and technology. In brief, vitality structures are a means to establish construct validity for ‘addictive’ and other psychologically relevant designs. Although the present work is primarily theoretical, I draw descriptive phenomenological data from my own experiences. Ultimately, I hope vitality structures can serve as a step toward more pragmatic and construct-valid approaches to explaining how people interact with contemporary and future technologies. The first sections outline important terminological clarifications, which are followed by analytical and theoretical scrutiny.

### On ‘Addictive’

I keep using marks around ‘addictive’ due to the still open research question about whether framing technology ‘addictive’ makes scientific sense (e.g., Billieux *et al.*, 2017; Ferguson & Colwell, 2020; Kardefelt-Winther, 2017; Sutton, 2020). Most experts—and especially non-experts—would agree that contemporary products that utilize the internet, mobile devices, and the computer are very efficient at capturing our attention continuously. From this viewpoint, it may be reasoned to call such technology ‘addictive’ just like good books, television series, and physical exercise can be ‘addictive’ in many ways (see Bell *et al.*, 2015; Billieux *et al.*, 2015; Brus, 2013; Calvo *et al.*, 2018). I should add that less controversial synonyms could be used here too, such as ‘engaging’, yet because the goal is to contribute to the clinical discourse, compromising with ‘addictive’ from a healthy critical distance feels justified. Further, we should remind ourselves that no substance, such as alcohol, is exclusively ‘addictive’ but simultaneously ‘engaging’, ‘tasty’, ‘stimulating’, and many other things.

In psychiatry, too, there has been increasing doubt toward the term ‘addiction’ and related terms nosologically (see Barry *et al.*, 2014; Botticelli & Koh, 2016; Broyles *et al.*, 2014; Matthews *et al.*, 2017). In the most recent *Diagnostic and Statistical Manual of Mental Disorders* (DSM-5-TR; 2022) the “more neutral term *substance use disorder* is used to describe the wide range of the disorder” (p. 543), covering drugs as well as gambling. However, the term ‘addictive’ remains in their chapter title and also the ICD-11 has a kept the word ‘addictive’ as a topic label for “disorders due to addictive behaviours” (for a recent perspective to classificatory challenges, see West *et al.*, 2023). Notably, this category is the one where also videogame-related health problems have their own formal diagnosis as *gaming disorder*.

Using the term ‘addictive’ with reference to videogame design and play can be tautological, nonetheless. For a design to be classified as such, it is necessary to identify the ‘addictive mechanism’, i.e. how a design pattern contributes to a type of addictive behaviour; while the addictive nature of the behaviour remains defined by the behaviour itself. I spell out the two key problems.

First, a construct-valid search for ‘addictive’ designs entails someone being ‘addicted’ to certain designs. Being able to say “ *that* gaming is/isn’t addicted” is a premise for outlining any (non) ‘addictive’ design, and we need to agree upon what ‘addictive’ is to identify relevant designs. For example, it is unclear whether ‘addictive behaviour’ should equally apply to someone being distracted by checking their smartphone regularly (e.g., Conroy *et al.*, 2023; Vainio *et al.*, 2023), and another person playing a massive multiplayer online game for decades (e.g., Bleckmann & Jukschat, 2015; Domahidi & Quandt, 2015). Should ‘distractive design’ be separated from ‘addictive design’? Although we have no space to answer such questions here, being aware of them will help us move forward.

Second, delineating an ‘addictive’ design *per se* as, say, alcohol or nicotine remains a holy grail in the field; also in the sense that we do not know if it exists. As Panova and Carbonell (2018) note, “smartphone addiction,” as a term, is the same as “drinking glass addiction” would be for alcoholics—identifying specific ‘addictive’ elements in design remains a challenge both methodologically and theoretically. For instance, one could propose the ‘red dot’ appearing in many smartphone applications as one type of ‘addictive design’ but such delineation would hardly be sufficient. The ‘red dot’ would need to be contextualized in the larger design framework of notifications (i.e., if and how it is different from other notifications) and applications where it occurs. When sufficiently contextualized and still deemed relevant, the connection between that construct and a user’s experiential engagement with it should be demonstrated. This can produce a hypothesis of the underlying clinical mechanism(s) that explain how a person ‘addictively’ interacts with it. Taken together, the present scenario can be described as one of “epistemic iteration” ( Chang, 2004) where successive stages of scientific knowledge are being built on imperfect preceding ones. His example of scratched glasses is helpful here:

*Without wearing my glasses, I cannot focus very well on small or faint things. Therefore if I pick up my glasses to examine them, I am unable to see the fine scratches and smudges on them. But if I put on those same glasses and look at myself in the mirror, I can see the details of the lenses quite well. In short, my glasses can show me their own defects.* (p. 230)

Although we do not have a good understanding of ‘addicted’ gaming nor do we even agree whether ‘addicted’ behaviour is even the right framing, we can use the state of art as a stepping stone without commitment. To make progress, we need to anchor the present gold standard of ‘addictive behaviour’ on the best available data. This will serve as an auxiliary hypotheses, which can later be corrected or rejected in the light of better data. Currently, the best data come from actual treatment-seekers who have problems with gaming. I return to this in the section dedicated to methods.

### On Design

Next, I address the problems that concern research on design structures. Identifying such structures as constructs that can be re-identified across different videogames (and non-videogames) is a challenge both methodologically and theoretically. To illustrate the range of these challenges, earlier design and psychological literature need to be consulted.

I find the research on the design of gambling games as the most fruitful starting point for two reasons: ‘addictive behaviours’ related to gambling are qualitatively well documented over several decades (e.g. Schull, 2012), and the designs that engage problematic gamblers have been studied longer than videogames have ever existed. Assumedly, at least the designers of gambling games know several mechanisms that have a high probability for contributing to people’s gambling behaviours. It was B.F. Skinner himself who suggested no later than in the 1950s that conditioning had already been efficiently integrated to gambling design, as the “efficacy of such schedules in generating high rates has long been known to the proprietors of gambling establishments” ( 1953/1965, p. 104). I consider Skinner’s insights valuable because they infer directly from theory, and theoretical failures, too, contribute to scientific progress. For example, Skinner very clearly described what is nowadays known as “near miss” as follows:

*Gambling devices make an effective use of conditioned reinforcers which are set up by pairing certain stimuli with the economic reinforcers … “almost hitting the jack pot” increases the probability that the individual will play the machine* (p. 397)

For good reasons, we thus see careful in-depth investigations of near-miss already in the 1980s (e.g., Reid, 1986). Alas, up to the present day, it remains unclear whether near misses actually have any effect and what potential kind. A recent failed replication review ( Pisklak *et al.*, 2020) persuasively suggests that, for half a century, “near-miss research may have been misguided from the start” (p. 629), and perhaps the psycho-phenomenological construct of engagement due to near-miss design manifests only in a yet-unidentified limited contexts and/or “as initial machine selection or amount bet” (p. 627; however, notice that this is what Skinner originally proposed but many researchers ended up modifying the operational effect of the mechanism!). In other words, the construct does not seem to be clearly enough understood yet to produce replicable experiments—if the null hypothesis is not true.

It is important to add that some such constructs seem to have more evidential support. For example, designing for “illusion of control” as letting players win more frequently at first may indeed increase players’ belief in winning ( Eben *et al.*, 2023a). Although a related effect of post-loss speeding seems to have support too, the underlying illusion of control construct remains inconsistent and leaves open the question of how its ‘designs’ interact with gamblers (see Eben *et al.*, 2023b). Moreover, from a phenomenological viewpoint, we know that many gamblers who identify their play as ‘addicted’ do not even care about winning—as says Mollie, one of Schull’s (2012) addict-identifying interviewees: “The thing people never understand is that *I’m not playing to win*” (p. 2). Even if players, under an illusion of control, believe more in winning, why would that matter if *they do not care* about winning in the first place? More than four decades ago, Loftus and Loftus (1983) proposed rapid replay as a means to eliminate regret, which remains a strong psychological candidate for post-loss speeding without illusion. Verified links between quantitative effects and qualitative experiences continue to be complex and difficult to draw.

Things get even more complex in videogames. Whereas all gambling is based on relatively simple design mechanisms involving random rewards—and gambling products are highly accessible in general due to their economic vector (see Karhulahti & Grabarczyk, 2021)—the most played videogames like *League of Legends* and *World of Warcraft* are extremely multifaceted and highly dynamic design products. Despite the fact that constructs like ‘near miss’ and ‘illusion of control’ might be possible to identify as small particles of such games, evidencing their effects in such context remains a challenge. A possible methodological alternative would be to deconstruct entire videogame designs or their segments to identify central patterns; however, creating a system for classifying different designs remains a meta-challenge that keeps hindering progress in this regard.

A decade ago, I made a still-relevant distinction between ‘augontological’ and ‘typontological’ approaches to the analysis of videogame structures ( Karhulahti, 2015). In the former, the idea is to map out all prevalent or relevant particles of games, such as design patterns and elements. For example, both Game Ontology ( Zagal *et al.*, 2005) and Gameplay Design Patterns ( Björk & Holopainen, 2005) collections include an entry called Scores, which represents a central element of design that can manifest in various ways across different videogames (and non-videogames). There is literally an infinite number of concepts like Scores and such collections can thus be expanded infinitely—there are currently 623 gameplay design patterns officially listed ( http://virt10.itu.chalmers.se/index.php/Main_Page). Some of these patterns (e.g., Grinding) are classified as ‘dark’ ones ( Zagal *et al.*, 2013), as they possibly contribute to negative gaming experiences (e.g., artificially increase expected play time).

Typontological approaches, in turn, do not aim at covering all elements but rather the dimensions by which such elements are built. For instance, in the typology by Elverdam & Aarseth (2007), there are no elements such as Scores but rather a dimension of Struggle that is divided into Challenges and Goals, and the latter further split into Explicit and Implicit goals. A score design could thus be an instance of an Explicit goal. Again, it would be possible to further expand a dimension like Struggle or Goals with an infinite number of additional subdimensions. As different research questions entail different degrees of depth, choosing optimal accuracy remains a pragmatic matter.

It is fair to acknowledge that the clinical research domain has addressed videogame design as well. For instance, King & colleagues (2010) have proposed a list of fundamental ‘psycho-structural’ elements of videogames to be applied in the study of mental health. The list consists of five main categories (social, manipulation, narrative, reward, presentation) that were largely self-selected with the guidance of gambling research (see Wood *et al.*, 2004). Notably, despite their intended clinical application, such lists do not derive from actual cases of health problems that manifest with specific videogames. In consequence, they do not have the depth of augontological and typontological systems, but also lack the clinical dimension.

Finally and recently, Flayelle *et al.* (2023) have approached technology analysis from a point of view that connects specific psychological mechanisms to potentially problematic design features. For example, affective mechanisms include processes such as emotion dysregulation, which in interaction with design can contribute to impaired control. Meanwhile, Meier & Reinecke (2020) have suggested a distinction between channel and communication centred approaches to health research on social media: the former focusing on design (application, feature, etc.) and the latter on how the design is interacted with (configuration, directionality, etc.). Although both the above studies do not investigate specific psychology-design combinations but review previous literature, such dual approaches are relevant to the present work and I elaborate on these links in the discussion section later.

### Vitality Structures

My philosophy of science is pluralist and pragmatic ( Chang, 2023), that is, I believe multiple competing programs of knowledge can exist at the same time as long as they all contribute to operationally coherent practices. It can be useful to keep running better experiments for expected design effects and building better (or bigger) classification systems of design—while at the same time, I believe none of these efforts alone or separately are sufficient to capture what is needed to understand ‘addictive’ videogame design, whatever that comes to mean in the future. My diagnosis of the issue is that related discussion often takes the ‘design’ as a starting point—i.e. a certain type of common design element—but fails to acknowledge the phenomenological (not merely behavioural) counterpart that connects to it. What we should care most is the human experience: not asking “what does a *design do*” (design → human) but rather “how did a person end up *feeling that*” (design ← human)? I aim to follow the latter epistemic sequence below. ^1^

My proposed design-phenomenological framework is motivated by the idea of ‘vitality affects’, as coined by developmental psychiatrist Daniel Stern in the early 1980s. Notably, the concept has developed over four decades ( Koppe *et al.*, 2008) and there is no one ‘true’ definition or interpretation of it. Koppe *et al.* (2008) offer a conceptual review of the concept’s history and summarize the initial form as follows:

*Vitality affects are connected to vital life processes such as breathing, becoming hungry, falling asleep, waking up, etc., to which the term ‘‘vitality’’ refers. They are, however, also present in principle in the carrying out of any kind of goal-directed mental activity, for example a thought progression, an interaction, or a dialogue. Stern exemplifies [these] vitality affects via transitory qualities: they are something ‘‘surging,’’ ‘‘fading away,’’ ‘‘exploding,’’ etc* ( Koppe *et al.*, 2008, pp. 170–71).

The authors suggest that some of the examples, such as vital body rhythms (breathing), should not be considered features of vitality affects because they tend to confuse the concept with general feeling(s) of a body. Instead of being associated with natural bodily functions, the conceptual focus should be on amodal, relationally felt aspects such as changes in direction, speed or intensity over time. I believe this is a useful criticism, so let us keep that in mind.

Two years before Stern passed away, he published a book titled *Forms of Vitality* (2010a) where the conceptual target moves from ‘affects’ to ‘forms’ (of vitality). Forms of vitality are more clearly defined in relation to dynamic events. Stern names five elements of which these dynamic forms consist: movement, force, space, diction/intention, and time. The reference to entities of physics is not a coincidence (he mentions Einstein’s description of ‘thought’ as inspiration). I cite Stern at length:

*I want to suggest that there is a special way that we experience dynamic events. Now when I say dynamic events, what I mean is events that unfold over time, that have a force apparently within them or behind them, that are going somewhere – these events, and that they seem to be pulled towards some goal. The important thing is the event seems to consist of some kind of movement, and it takes time, and it also occurs in some kind of space, even if it’s mental space... I am going to call them Forms of Vitality* ( Stern, 2010b, p. 88)

This is where I pause and hope you have a tentative picture of what I have in mind when using the term ‘vitality’. To be clear, I am not interested in debating about the ‘true’ nature of vitality as expressed by Stern or someone else; rather, my goal is to communicate a certain type of vitality, which I learn from Stern’s description: a feeling of (not random but) directional/intentional movement in mental space that has some force, and this movement naturally manifests over time as we experience it. These are dynamic qualia, which are present almost every moment in our lives as we are awake. For example, drinking from my coffee cup involves numerous ‘forms of vitality’ from the specific “touch of the cup” (warmth that starts with short contact and expands) to the feeling of “coffee running out of the cup” (‘lessening’ in a sense that there is less and less of it as I drink), and to the feeling of “last sip” when I empty the cup (void after the fact). In all these small examples, different types of movement with force occur in space through time and they have certain ‘intention’—not in a sense that it would be *my* intention or action, but the force has its own intention as ‘directed’ or ‘determined’ course, as when the coffee “runs out.”

From the above, I derive *vitality structures* as ‘bonds’ between technology and human experience—theoretically warranted constructs, the long-term validation of which is a matter of both design and phenomenological experiments. I should clarify that vitality structures are not only features of design, nor merely something experienced. They are constructs that bond *specific* designs with *specific* experiences. Although we may use vitality structures as reference to either the design-side or the phenomenology-side of the construct, it is important that neither exists without the other. The ‘structures’ (of vitality structures) highlight the dual nature of the design-phenomenological framework: it is a ‘structured’ experience, as specific videogame (and non-videogame) designs can be built to provoke the experience, but the design alone is not a vitality structure until we identify the corresponding experience. It is possible to interact with the design-side of an identified vitality structure *without enacting the corresponding experience*, and it is also possible to have a similar experience accidentally in a different life context. It is the bond between a design and its phenomenological correspondence that delineates vitality structures as dualities.

**Figure 1.**
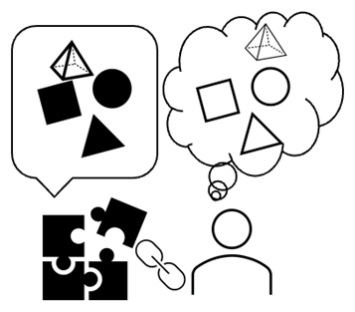
A videogame involves vitality structures by design, and players attune to such vitality structures by playing. Author’s own graph, no further permission needed.

I want to avoid overly metaphors, but one will aid us to better grasp what is being discussed. For Stern, vitality surfaced at first in the infant-mother relationship (see Ammaniti & Ferrari, 2013; Modell, 2018) where the two have no shared language yet, but they can feel or sense what the other is feeling or sensing by perceiving corresponding vitality forms and (re)enacting them. Stern calls this *affect attunement*. By reacting to and with gestures, sounds, and other means, the amodal vitality forms can be conveyed from one human to another. My intention is not to anthropomorphize technology, but I believe it helps to think about the human-technology relationship in a similar way: non-speaking applications like videogames conveying their players ‘vitalities’ that have no corresponding words but ‘forms’. Stern (2010a) would have agreed with this metaphor, as his very idea was that “vitality forms are readily transferable between art forms” (p. 79). When people play videogames, a type of affect attunement takes place: players attune to specific forms of affective engagement that someone designed ( Figure 1).

The attunement of vitality structures also reminds us of John Dewey’s (1934) classic theory of artworks as ‘experiences’ that manifest when people interact with artefacts that bear aesthetic qualities. Although a product of art has an increased probability to trigger a (specific) aesthetic experience in a person, aesthetic experiences may occur also as the “zest of the spectator in poking the wood burning on the heart and in watching the darting flames and crumbling coals” (p. 3). Likewise, a certain ‘addictive design’ may be prevalent in videogames, but its corresponding vital experience can also be unintentionally experienced elsewhere. It is important to stress that what I have referred to as a ‘corresponding experience’ is naturally a very empirical matter: to what degree people’s experiences of an identified vitality structure actually correspond with a design becomes a research question to be investigated and replicated. Yet before that, good hypotheses are needed. I set out three hypothetical vitality structures for testing.

## Methods

A recap is in order. The first challenge of studying ‘addictive designs’ is to know what ‘addictive behaviour’ related to a design is; only with a good auxiliary hypothesis regarding a certain design being involved in ‘addictive behaviour’ can we identify such designs. The second challenge is to effectively delineate a design and its underlying clinical mechanism(s). Merely naming a common design feature is not useful, but it needs to be identified as an applicable construct with sufficient accuracy and its links to people’s experiences thereof properly outlined. In this way, we can move toward hypotheses of selected designs to be tested for their ‘addictive’ nature across different contexts.

To tackle the first challenge, my basis for the present work shall be a list of videogames played by actual treatment-seekers in Finland. In our earlier research ( Karhulahti *et al.*, 2023a), we studied the reasons of gaming-related treatment-seeking with 110 participants who had applied for local clinical services. The participants named 26 unique videogame titles more than once as sources of their problems, and the examples to which I will refer come from these titles. Importantly, we currently lack in-depth data regarding *how* the participants experienced specific titles as problematic. Therefore, the vitality structures that I draw may not fully correspond with the problems experienced by those individuals, and this will need to be tested separately in other studies.

As has become clear, my proposed solution to the second challenge is a vitality structures approach where the delineation of a construct flows primarily from human to design, and is iterated in a design-phenomenological framework. To access the phenomenological domain and be able to identify potentially relevant ‘addictive’ designs, I refer to my own experiences as a player of three videogames, which multiple treatment-seekers named as sources of their problems. The method is not ethnographic but retrospectively reflects my ‘organic’ player experiences in the past. I do not consider these experiences ‘addicted’ but highly ‘intensive’ nonetheless. Arguably, this makes me imperfectly but still well positioned to identify potential ‘addictive’ designs in these titles. I disclose further details about the titles below.

***League of Legends (LoL)*** is a team esports videogame that represents the so-called MOBA (multiplayer online battle arena) genre. Matches are played in 5-player teams and last approximately 25–30 minutes. I played LoL competitively for years (approximately 3,000 hours), details of which are documented elsewhere ( Karhulahti, 2020). After quitting in 2018, I have followed the game’s evolution from a distance. According to the game’s open API, I have nonetheless played 136 hours in the last five years. Recently, I have also played the almost identical mobile version *Wilf Rift* (WR) with some competitive motivation, yet with more modest efforts (approximately 200 hours overall).

***Clash Royale (CR)*** is a competitive mobile card game. Matches are played 1v1 in a tower defence setting, and they last 2–5 minutes. Because success in CR is partly based on card strength that can be maximized only by continuous micro transactions, my competitive motivation is limited (I am against pay-to-win designs in principle). I have played CR since 2018 as a daily pleasure. According to the open API, my lifetime play investment is 781 hours. The game is designed to encourage frequent daily log-ins, so I play a few times per day (about 20 minutes in total).

***Soulsbornes (SB)*** are single-player action roleplaying storygames, which also have optional multiplayer modes. The series is known for its high difficulty and includes six independent instalments, which are mechanically almost identical: *Demon’s Souls* (2009), *Dark Souls* (2011), *Dark Souls 2* (2014), *Dark Souls 3* (2016), *Bloodborne* (2015), and *Elden Ring* (2022). I completed all titles once and each took about 100 hours of playtime, thus I have spent about 600 hours on Soulsbornes. I did not engage in their competitive multiplayer modes, albeit some of them enforce occasional engagement with other players.

In the analysis, I will utilize the above histories of engagement to propose three vitality structures that appear frequently in one or more of these videogames. The conceptualisations of these vitality structures and their phenomenological relevance for intensive gaming have evolved in the past decade introspectively; I have no other method for their development. I do not have evidence nor do I claim that the chosen vitality structures are the most central in terms of ‘addictiveness’. Rather, I have chosen them because they represent a good variety of differently relevant vitality structures and serve as helpful illustrations for the current primer.

## Analysis

Vitality structures are not natural kinds. By this I mean that each vitality structure is an abstract representation of mental movement: what people feel or sense when interacting with a design. Two feelings can never be exactly the same, and there is usually some overlap with two different feelings. This does not mean that vitality structures cannot be studied empirically or they have no empirical basis; quite the contrary. A good vitality structure should empirically resonate with the experiences of multiple people, and this correspondence should be validated by multiple empirical methods. The present autographic reflections are one such limited method, and a beginning for development.

For practical purposes, I see vitality structures in macro, meso, and micro *chronotopes*. By chronotope I refer to experienced spacetime; both the ‘size’ and ‘length’ of the experience, as further explained later. Naturally, the three chronotopes are not clearcut but represent a continuous spectrum. A macro-chronotopic vitality structure is felt via ‘expansive’, ‘prolonged’, and ‘slow’ mental movement. A meso-chronotopic vitality structure is felt to be ‘faster’ and more ‘intense’, and a micro-chronotopic one is ‘immediate’ and ‘sharp’ as if experienced in the very moment. I understand that my words cannot fully communicate the intended qualia. I hope the upcoming examples do better. Before that, it is important to note that a vitality structure can manifest as one, two or all three chronotopes: a micro-chronotopic ‘structure’ may sometimes be extended into a meso- and macro-chronotopes, and vice versa.

The chronotope involves both spatial and temporal description, but each can be further expanded. I will thus add two meta-dimensions across which vitality structures can extend: *meta-spatial* and *meta*- *temporal*. By meta-spatial I mean whether the vitality structure is experienced ‘close’ to the player’s self or farther away, as through an avatar or object. Examples will clarify this soon. By meta-temporal I mean whether the feeling is ‘as if one looks back’ at something (‘having moved’), is in the very process of that mental movement at the very time (‘moving now’), or anticipates it in the future (‘will move there’). This dimension, too, will be easier to grasp soon. Both dimensions can be looked at through the distinction between ‘minimal’ and ‘narrative’ self: sense of vital self-agency or ownership experienced as extended or being devoid of temporal and spatial extension (e.g., Gallagher, 2000). As we shall see, whether a vitality structure can expand to all three (meta)dimensions depends on the nature of the specific structure.

As a final note, I will follow Jaak Panksepp (1998) and use capital letters for the proposed vitality structures to avoid them being directly associated with the culture, etymology, and meaning of my chosen label words. Although I have tried to find labels that give accurate phenomenological inference about what I am trying to express, it is essential to acknowledge the burdens and limits of language when communicating human experience. In this regard, our topic is not so much different to Panksepp’s attempts to describe affective sensations across animals.

### CLIMB

CLIMB refers to a dynamic feeling of ‘going up’ by means of exerted effort. It overlaps with the general sensation of ‘progress’—which is felt as a *forward*-directed force—yet CLIMB has at least two distinctive features. First, whereas progress may happen automatically by its own (feeling of story progress; moving onwards in space; etc.), CLIMB entails exerted effort that represents an *upward*-directed force. When a player experiences CLIMB, it feels as if “I’m making it” and not merely passively moving to a direction. Common designs that trigger CLIMB in players are the ranked ladders in esports (the ‘ladder’ metaphor is not accidental) and level-up systems in roleplaying games (the post-fix ‘up’ is not accidental).

**Figure 2.**
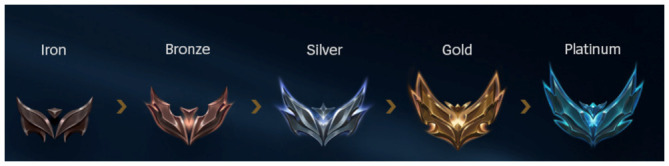
The first half of ranks in LoL. Author’s own screenshot, no further permission needed. © Riot Games.

In LoL and WR, the ranking systems are nearly identical and success is generally referred to as climbing in their player communities. Winning matches accumulates points that eventually lead to higher ranks. Both systems are also visually represented as increasingly *higher* or *taller* ( Figure 2). In these systems, CLIMB is designed and experienced macro-dimensionally: one, two, or tree victorious matches do not lead to any change yet, as even the smallest step upward usually requires five or six consecutive wins, i.e. several hours of playtime. More often than not, players cannot win consecutively and reaching the next tier takes several days, weeks, or months. Therefore, experiencing CLIMB via the ranking systems of LoL and WR feels ‘sluggish’, gentle motion in the background until occasionally manifesting from a horizon. CLIMB can be sensed from three different meta-temporal perspectives—retrospectively, prospectively, and in the present—as if “I made it all the way up here” (looking down), “I’m going to be there” (looking up), and “I’m getting there (an immediate feeling when *taking* a step).

CLIMB can be experienced similarly in CR. Currently, the mobile game provides players with two separate ladders, Path of Legends and Trophy Road, which are both presented as ladders to be climbed up. By winning, players earn points that will move their position on the ladder upwards. The former ladder resets season by season (approximately once a month), which makes experiencing CLIMB through it ‘faster’ or more ‘intense’—even a single victory (of a 2–5 minute match) can lead to a step up of one stair. Movement in the latter, in turn, requires several victories as in LoL and WR. As such, experiencing CLIMB in Path of Legends could be described meso-dimensional: a background feeling that is not immediately present yet surfaces right after each match. Notably, the design is structured to often produce CLIMB by a forced visual animation of upward movement as a post-victory cutscene; meanwhile, a post-loss movement downwards is left uncommunicated (to avoid activating negative vitality structures, perhaps).

In SBs, in turn, CLIMB is primarily designed as a level-up feature: players collect currency that can be spent to level-up their avatar. Here CLIMB is not connected to the player itself but their avatar in control, i.e. experienced more distantly in the meta-spatial dimension. As is typical for roleplaying games, level-ups also increase stats, which players can freely distribute; the link between increasing stats and CLIMB together accumulate empowerment (arguably a separate vitality structure; see Linderoth, 2013). In addition, SBs also allow players to level-up equipment, such as weapons and talismans. Equipment CLIMB, again, feels even further distanced because it is not ‘you’ or your representation as avatar, but rather something you have—an external object. Even though the upward-directed experience is separate from one’s body, the structure of CLIMB remains similar.

In Table 1, I present the phenomenological spectrum of CLIMB through the previously introduced three dimensions. Although more dimensions and their continuous degrees can be added—each vitality has an infinite number of possible manifestations—the present 27 variations of CLIMB should be pragmatically sufficient for illustrating how it can be differently experienced in videogames (and non-videogames).

**Table 1.** Three-dimensional variation of CLIMB. Meta-temporal dimension presented as Past/Present/Future.

META-SPACE / CHRONOTOPE	Close (self)	Distanced (e.g. avatar)	Far (e.g., object)
**Micro** (tiny/instant)	Past/Present/Future	Past/Present/Future	Past/Present/Future
**Meso** (smaller/faster)	Past/Present/Future	Past/Present/Future	Past/Present/Future
**Macro** (large/slow)	Past/Present/Future	Past/Present/Future	Past/Present/Future

### FINAL STRECH

FINAL STRETCH refers to a specific feeling of a yet-unfulfilled goal at reach, which requires a relatively small effort to be finalized. It is the moment ‘just before’ something is completed or finished, and importantly, one explicitly feels “I could or should complete that” and not only “it’s close” or “it’s getting closer”. One feels a ‘pulling force’ toward closing the gap, as something that is feasible and a ‘sensible’ thing to do (sense referring to both bodily and noematic momentum). A recurring FINAL STRECH surfaces with CLIMB in level-ups and ranked ladder: as players are about to reach the next level or tier (“just one more win to get there”), they feel a need for ‘completion’ or ‘closure’ that will settle the project momentarily. However, this is just one of the many possible contexts where FINAL STRETCH can occur. For instance, videogames that involve collecting items or progressing a story may likewise trigger FINAL STRECH (as in ‘cliffhanger’). Unlike CLIMB, the phenomenological structure of FINAL STRECH does not seem to bend usefully to a past meta-temporal perspective, as the ‘gap’ ceases to exist after it has been closed. Therefore, I only list the present and future meta-temporal dimensions ( Table 2).

**Table 2.** Three-dimensional variation of FINAL STRECH. Meta-temporal dimension presented as Present/Future.

META-SPACE / CHRONOTOPE	Close (self)	Distanced (e.g. avatar)	Far (e.g., object)
**Micro** (tiny/instant)	Present/Future	Present/Future	Present/Future
**Meso** (smaller/faster)	Present/Future	Present/Future	Present/Future
**Macro** (large/slow)	Present/Future	Present/Future	Present/Future

**Figure 3.**
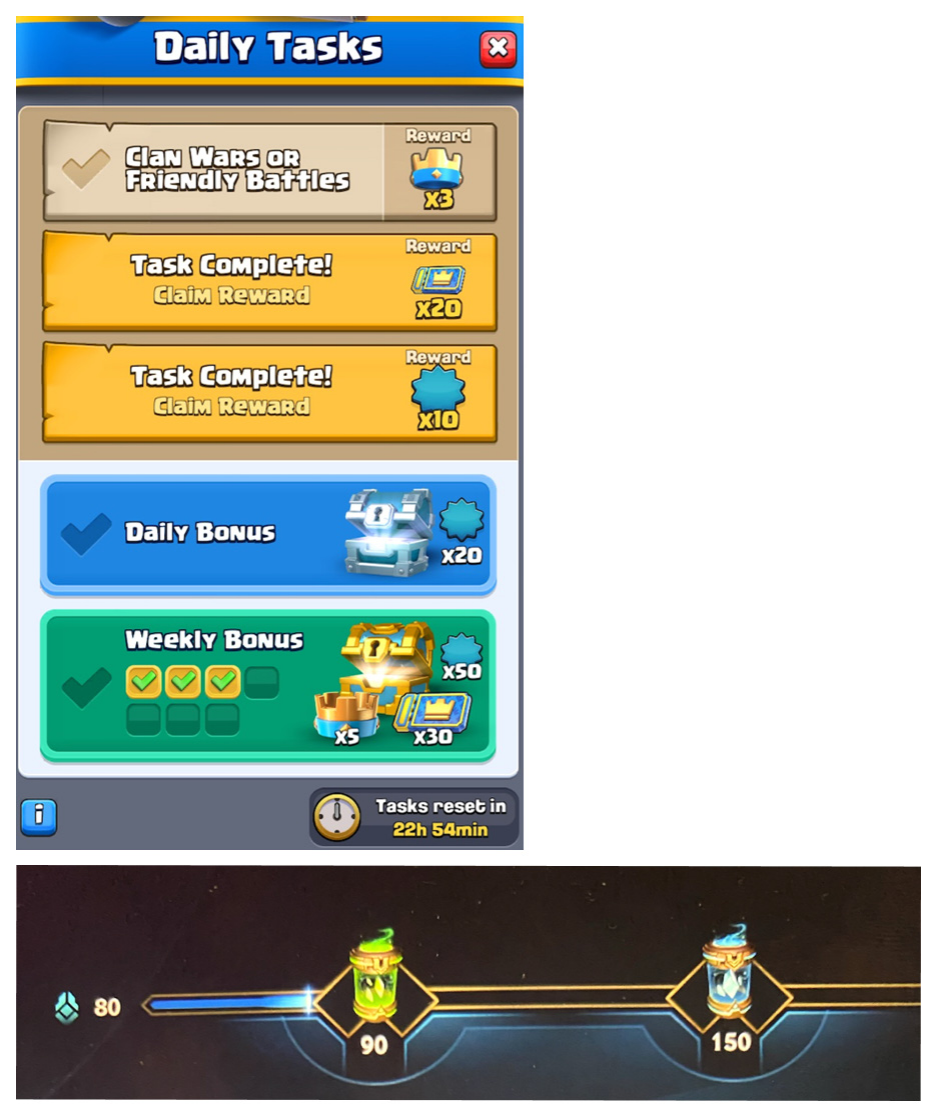
After logging into WR, the first daily capsule is almost at reach (below). After logging into CR, the daily bonus is almost at reach (above). Author’s own screenshots, no further permission needed. © Supercell and © Riot Games.

As FINAL STRECH motivates the player to keep playing, many videogames involve related design structures. In WR, the opening home screen currently rewards players (every day) with two return prizes that accumulate the player’s daily activity points to 80, which is close to earning the green capsule that opens at 90 points ( Figure 3). The feature communicates that reaching the green capsule is near and by playing just one match, the player *will certainly* reach the green capsule. The same reoccurs if the player loses a match: a loss will grant enough points for the green capsule but leaves the blue capsule (at 150 points) out of reach, i.e. the player needs to play one more match. I recall many times when my plan was to play one match, but FINAL STRECH surfaced after a loss and led me to play another match to reach the valuable blue capsule. In all these cases, the FINAL STRECH is felt near ‘self’ in the meta-spatial dimension.

CR utilizes a similar design. The daily rewards on the home screen are based on task activity, and usually two of the three required tasks are possible to accomplish without playing a single match (e.g., visit the shop and open a reward chest). As the third task conventionally requires playing a match ( Figure 3), opening the CR application and collecting directly accessible rewards is clearly designed for FINAL STRECH, which induces play. This vitality structure, too, operates throughout multiple chronotopic degrees so that smaller and immediate projects are nested inside larger and longer projects: the first reward is only a single click away, the daily reward is only a single match away, and the weekly reward is six days away (and gets closer every day). Moreover, this is but a single form of FINAL STRECH in CR, as other features such as ranking ladders and the Daily Event promote it too. Currently, players can earn up to 1000 season tokens per day by playing in the Valhalla event; the cap is almost reached by one win, but even five losses are usually not enough to collect all daily available tokens. Experiencing FINAL STRECH can keep players (like me) playing to reach the cap, which can be simultaneously far and close (depending on the win rate).

Immediate and small types of FINAL STRECH manifest in LoL, WR, and CR. During each match, players control various characters; these characters increase their strength by level-ups, which contribute to their temporary empowerment over time. For example, during a 30-minute match of LoL, the player’s chosen champion can be level-upped 17 times, with several additional upgrades related to equipment. As such, a 30-minute match can involve several instances of FINAL STRECH when the player feels a need to collect the last bit of experience or gold to reach the next level or equipment update (with the caveat that it can also happen unexpectedly). These ephemeral and instantaneous moments accumulate, until the match ends and all character stats reset—to be reacquired in the next match. By creating such parallel and nested caps, goals, and tiers, designers can incite players re-feel FINAL STRECH several consecutive times on different experiential dimensions.

Unlike the above, the design structures of SBs rarely provoke FINAL STRECH. However, there are instances in SBs where FINAL STRECH can manifest organically. In my own experience, for instance, being close to reaching the next level-up would often trigger FINAL STRECH and I typically continue playing until reaching that milestone. Arguably, any design that slices progress, rewards, or other anticipation channels into small milestones contributes to FINAL STRECH by creating the impression that projects are close to completion.

### ALERT

This vitality structure could be named ‘note’ or ‘notification’, but I have chosen to call it ALERT to highlight the active momentum and be more inclusive to various ways in which our attention can be alerted to events. Essentially, ALERT is felt as something ‘immediate’ or ‘instant’; it is ‘acute information’ in a sense that one knows it is ‘there now’ and there is a way to access that information. In particular, ALERT refers to that ephemeral and short feeling that rushes through when a player becomes aware of such a signal. Even though conditioning can be part of many ALERT vitality structure forms, it is not simply an activated Pavlovian neutral stimuli paired with a reward, but a felt ‘spike’ or ‘shock’ that has a dynamic form: one feels a need to investigate and reduce uncertainty by instant exploration.

Whereas the structure of CLIMB was previously described as highly flexible in chronotopic, meta-spatial and meta-temporal dimensions—and FINAL STRECH in chronotopic and meta-spatial dimensions without a past-oriented perspective—the structure of ALERT extends meta-spatially but is fixed to a micro-chronotope without a past-oriented perspective ( Table 3). The phenomenological differences between present and future perspectives can be described continuously from ‘doing it’ to ‘postponing it’; or, a positive ‘surrendering’ response and a negative ‘resisting’ response. In the latter, one feels the dynamic pull that is characteristic to ALERT but counter-pushes or suppresses it in a way that the vital movement slows down or stops. Nonetheless, the nature of ALERT is essentially micro-chronotopic—it is difficult to imagine ALERT being ‘lasting’ or ‘prolonged’ but the feeling is always instant even though it can be postponed to be instantly re-experienced later.

**Table 3.** Two-dimensional variation of ALERT.

META-SPACE / CHRONOTOPE	Close (self)	Distanced (e.g. avatar)	Far (e.g., object)
**Present** (“doing it”)	Micro (tiny/instant)	Micro (tiny/instant)	Micro (tiny/instant)
**Future** (“postponing it”)	Micro (tiny/instant)	Micro (tiny/instant)	Micro (tiny/instant)

During play in LoL, leveling up one’s champion periodically triggers ALERT by blinking lights that indicate an immediate opportunity to set new ability points. On the home screen, outside actual play, collectible rewards are communicated with highlighted buttons. These are strongly present especially in the WR mobile version, which also efficiently utilizes audio to ALERT players with interactable rapid information, such as friend and match invitations. In these contexts, ALERT serves primarily as a communicative or instrumental function: it informs players of their agency and options for interaction, and very few design features appear to provoke ALERT in intentionally manipulative ways. Only the first daily interactions with the opening home screen, as discussed earlier, involve features that potentially utilize ALERT to encourage returning players to start playing.

**Figure 4.**
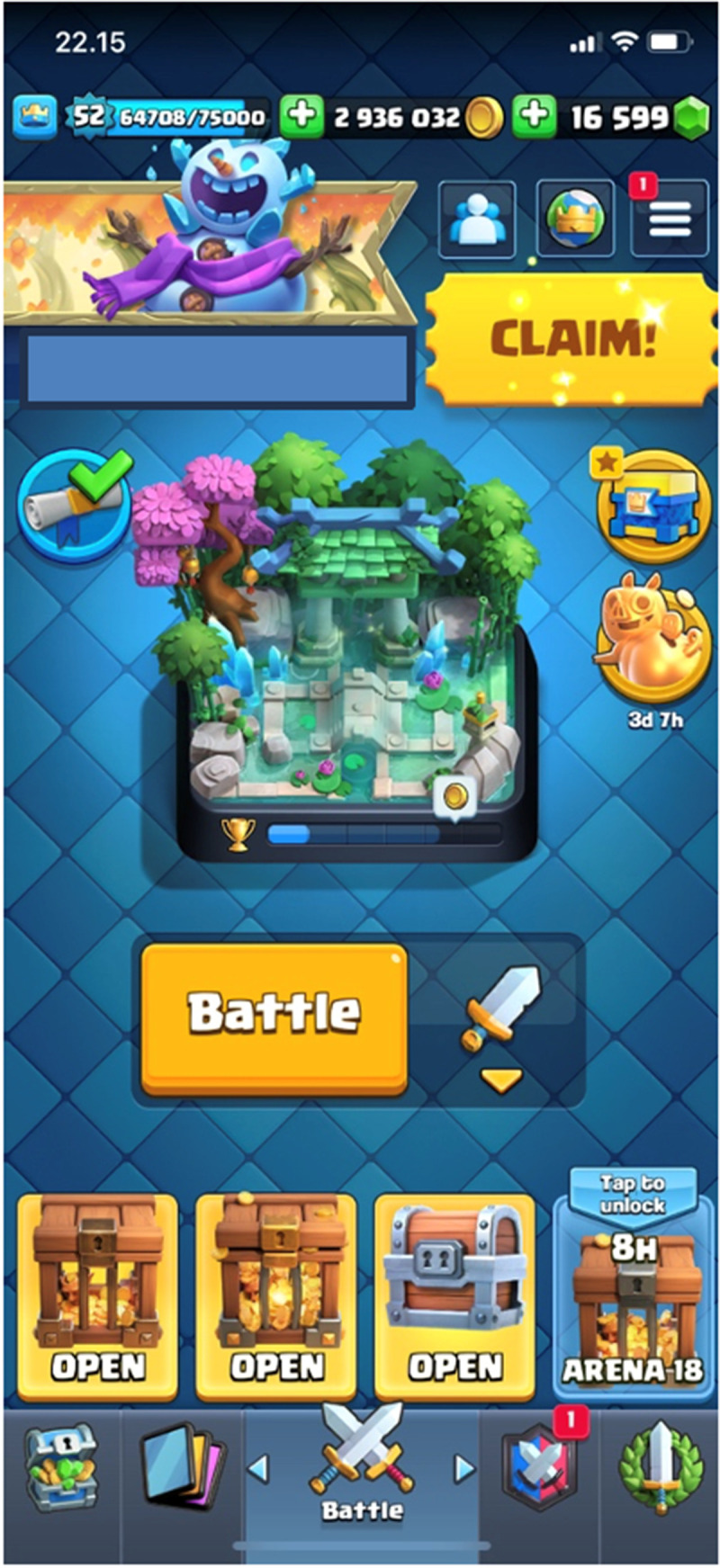
Home screen of CR with a dozen alerting design features. Author’s own screenshot, no further permission needed. © Supercell.

In CR, ALERT has a more central experience function that relates to its design as a mobile game and the expectation that players re-return to play for short periods, several times per day. Currently, my home screen ( Figure 4) shows three ‘shine-animated’ reward chests, one ‘beating’ Claim-button for pending progress rewards, one ‘jumping’ chest animation that implies an unused banner, one ‘pulsating’ encouragement to purchase Hoggy Bank boost for 2,49€, one static red mark that signals a pending friend request, and another static red mark reminding of the possibility to donate cards to a clan member. As this is a very typical number of ALERT-inciting design features for a regular daily log-in, it is clear that ALERT in CR is excessive. This is consistent with the game’s design orchestration: based on its 3-hour and 8-hour chest timers, an active player would open the application every 3–8 hours, i.e. at least 3–5 times per day. My subjective rhythm aligns with breaks during work hours and perhaps one or two additional sessions, which adds up to some 20 minutes per day divided into 3–5 sessions. Due to the shortness of these few-minute sessions, tiny ’shocks’ or ‘spikes’ of ALERT easily make a player (like me) click a few extra times per each session; this might not take more than a minute overall but these minutes may easily accumulate into a quarter of play time, i.e. a significant proportion of engagement annually.

In SBs, the design almost completely lacks ALERT features. Only the most essential game-relevant information such as ‘flask upgrades’ are communicated by alerting interface design—even highly important level upgrades have to be manually checked. The difference is consistent with SBs design orchestration: sessions typically last several hours because the progress structure requires lasting engagement. Although it is possible to play SBs in shorter sessions, the challenges are often chained and require time-consuming focus; being frequently alerted would unlikely contribute to the experience but rather distract from it. Compared to LoL, WR, and CR, a core difference is that SBs do not monetize by microtransactions. In the former, ALERT appears to be commonly designed for microtransaction promotion, i.e. special offers and new store items are merged among other ALERT signals to make players notice sales events. Evidently, the overall design constructions of videogames impact the utility of diverse vitality structures.

## Discussion

My goal has been to propose *vitality structures* as a design-phenomenological framework that can be applied to build construct validity for psychologically meaningful features of technology. I have described three potential vitality structures (CLIMB, FINAL STRECH, ALERT), which are part of videogames that have been involved in treatment-seeking. These limited examples demonstrate how different vitality structures can be both related and unrelated to each other, and how they can contribute to gaming experiences. At this point, Linderoth’s (2015) idea of game design as Goffmanian ‘frame orchestration’ is relevant: players frequently switch the ‘frames’ through which they interpret gaming-relevant events, and a core part of design is to orchestrate those frames. As I have shown above and will elaborate on below, there are dissimilarities, for instance, between the orchestration of MOBA, mobile, and single-player roleplaying games—and these variances not only rhythm gaming sessions but also their vitality structures.

**Figure 5.**
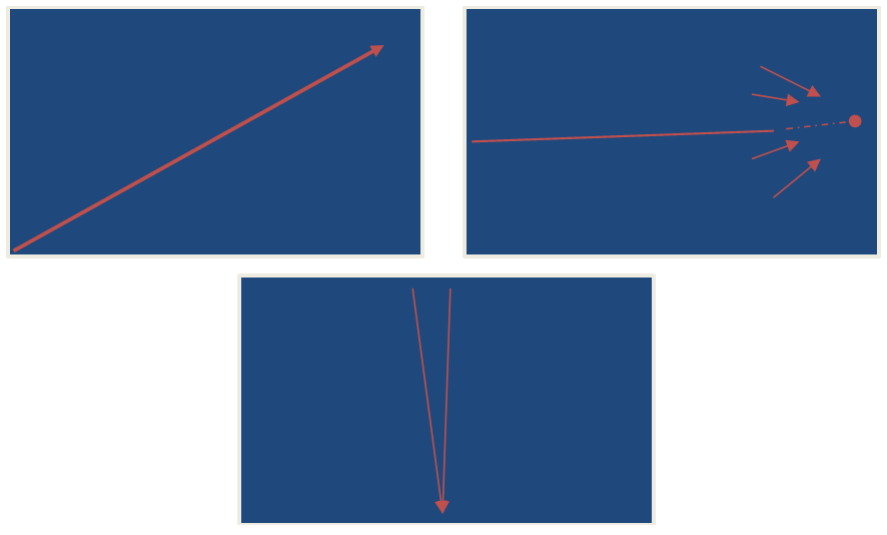
An attempt to visually represent the mental forms of movements in CLIMB (left), FINAL STRECH (right), and ALERT (bottom). Author’s own graphs, no further permission needed.

It is important to stress one more time that vitality structures are not exclusive to videogames, and similar features can be experienced in other life contexts. For example, people in academia may feel CLIMB in relation to their careers, FINAL STRECH when nearing the completion of a study, and ALERT as we receive work-related notifications ( Figure 5). The difference is, videogames and other technological products are explicitly *designed* to generate such vitality, and thus support as well as maintain these experiences—which obviously have proven efficient for controlling or aggregating user engagement. Vitality structures thus apply also to gamification, social media, and other such design efforts. In this regard, there is no unique ‘addictive’ substance in vitality structures; rather, designing for certain vitality structures optimizes what people experience naturally. To what degree such optimization is ethical or clinically hazardous should remain to be studied from various perspectives against potential benefits.

As highlighted earlier, vitality structures are not natural kinds and thus defining or identifying them is not a matter of truth but an effort of practice. There are no right or wrong vitality structures, yet it is possible to collect evidence for the utility of framing vitality structures in certain ways. With that in mind, I do not expect that my present framings of the three chosen vitality structures are final let alone flawless; they are based on how I have subjectively experienced the videogames in question (design ← human) and thus serve primarily as hypotheses. Plenty of earlier research have referred to similar phenomena, which contributes to my belief that we are looking at the right direction. I review some of that literature and address the applicability of the design-phenomenological framework.

### Earlier signs of CLIMB

Experiencing CLIMB has been briefly mentioned in various qualitative investigations of gaming-related problems. Already more than a decade ago, Nardi (2010) briefly mentioned level-ups in her ethnographic study on *World of Warcraft* (WoW) in relation to its ‘addictive’ qualities. In another monograph on the same game, Karlsen touches on CLIMB: “Different from gambling, World of Warcraft players are not grinding or questing because of the possible outcome of a random (and lucky) event, but because the outcomes, in the form of resources, act as stepping-stones to other goals” ( 2013). Snodgrass *et al.* (2014) too applied ethnographic methods to study WoW players and some of their accounts reflect CLIMB experiences, such as that of Derek:

*And so I was playing with my brother because he wanted to get up to as high rank as he could. For an entire summer we put in probably close to 8 to 10 h a day every day. And looking back on that summer, you know, it feels, it feels really kind of hollow. I spent some time with my brother, but it’s almost like a wasted summer, like one of the lost ones I guess in way.* (p. 255)

More recently, Snodgrass *et al.* (2021) also discuss climbing the ranks in *Counter Strike: Global Offensive* in the context of Indian players who self-identified as ‘addicted’ to the game. My own earlier anthropology ( Karhulahti, 2020) offers in-depth descriptions of CLIMB as ‘anticipation channels’, and in the phenomenological follow-up work with treatment-seekers ( Karhulahti *et al.*, 2022; Karhulahti *et al.*, 2023b) we found some participants—in this case Caius—explicitly describing CLIMB as a core source of the problems they had with videogames:

*It’s all about competitiveness [and specifically] climbing the ranks ... even in WoW, which didn’t have the ranking system, it had a third-party Elo system that enabled me to play it competitively [and] get addicted to climbing* (Caius in Karhulahti *et al.*, 2023b, p. 7)

As these examples show, CLIMB-related experiences have been documented in the past, yet without explicitly giving it identity as a construct. The psycho-structural framework by Wood *et al.* (2004) and its update by King *et al.* (2010), for instance, list “leader board features” as one of the numerous “social features”, i.e. they define the concept as part of sociality. From another general point of view, thousands of studies since the 1980s have mentioned ‘competition’ as a central part of videogames and their design (e.g., Malone, 1980). Alas, because all videogames can be played ‘competitively’, these accounts have little power to explain competitive design and related phenomenology unless further specified.

As a clinical hypothesis to be developed, I expect that people to whom videogame-related problems concern CLIMB have a significant probability for comorbidity by scoring high on measures of attention deficit hyperactivity disorders (ADHD) and autism spectrum disorder (ASD). A theoretical justification for these links is the established evidence for both disorder categories to phenomenologically involve hyper-level ‘immersion’, ‘fixation’ and/or ‘focus’ for activities that they care about (e.g., Baron-Cohen, 2008; Hallowell & Ratey, 2011). My assumption is that CLIMB—especially with long-term macro-chonotopic forms—is a vitality structure that efficiently connects to these ADHD and ASD human tendencies, both when qualified and non-qualified for a diagnosis.

### Earlier signs of FINAL STRECH

There appear to be few instances that dovetail FINAL STRECH in the previous literature. Arguably, the structure overlaps with self-efficacy ( Bandura, 1977) where one feels enabled to accomplish a meaningful task at reach. Similar ideas of goal completion have been discussed also in philosophical economics (e.g., Loewenstein, 1999), yet there are few empirical investigations related to it in the research programs on gaming. For example, even though a gambling mechanism like ‘near miss’ (e.g., Pisklak *et al.*, 2020) may look similar—as if ‘almost there’ or ‘almost a win’—it refers explicitly to the past with a certain ‘frustration’ or ‘regret’ afterwards. FINAL STRECH is a toward-pulling feeling that manifests in the present, and can be postponed to the future.

Another similar concept is the relatively much-studied ‘sunk cost’ effect (for a critical overview, see Friedman *et al.*, 2007). The idea of sunk cost is that one’s evaluation of future decision-making becomes biased by the costs (financial, temporal, etc.) invested earlier, e.g. it is more difficult to change a plan after one has already worked on it. There can be some conceptual and pragmatic overlap with FINAL STRECH and sunk cost when a player decides to complete a gaming task (or is inclined to do so); however, for the latter to take place, there should be an alternative ‘lost cost’ scenario that manifests in case of non-completion. For example, in all esports titles discussed earlier (LoL, WR, CR), interrupting a match that has started could logically produce one kind of sunk cost effect, yet this would hardly correspond with FINAL STRECH. On the other hand, a single-player videogame that allows saving a game state only at predefined locations might simultaneously support both FINAL STRECH and sunk cost.

In a comprehensive review of clinically relevant design mechanisms, Flayelle *et al.* (2023) taxonomize ‘partial goal fulfilment’ as a class of model-based features. In my interpretation, their model-based design generally refers to technological feedback without randomization. Design elements that incite FINAL STRECH could be defined as model-based under partial goal fulfilment. That said, it is important to keep in mind that vitality structures are not design elements *per se* but design-phenomenological constructs that cover a range of possible designs that correspond with an identified vitality. Following Meier and Reinecke (2020), it can be useful to design future research efforts on vitality structures to involve the very interactions carried out by the relevant player population.

My clinical hypothesis is that people to whom videogame-related problems concern FINAL STRECH have a significant comorbidity probability for scoring high on measures of obsessive-compulsive (OCD) and related disorders. Phenomenologically described as a need “to achieve a sense of *completeness*” ( Stein *et al.*, 2019), it would make sense that videogames designed for a ‘sense of incompleteness’—e.g., mobile genres orchestrated by rapid-frequent access rhythm, as discussed earlier—can provoke uncomfortable FINAL STRECH in people with OCD-related symptoms and tendencies. In particular, I see related problems manifest as prolonged gaming sessions by a need to complete ‘one more turn’ or overspending on gaming to receive completion satisfaction at any cost. Although further nosological discussion must be had elsewhere, such instances seem to follow a similar logic as “Obsessive-compulsive or related disorder induced by other specified psychoactive substance” (ICD-11; 6C4E.72) where underlying OCD symptomatology would be activated not by substances but technology use.

### Earlier signs of ALERT

As demonstrated in earlier analysis, in many videogames ALERT appears to serve minor communicative functions. The mechanism of these functions can be partially explained by the predictive processing approach to videogame design ( Deterding *et al.*, 2022), namely, players reducing uncertainty by responding to design features that offer low-cost-high-information value. Although ALERT can be a central part of mobile gaming that operates with high frequencies and short engagement spans, most examples in previous literature seem to come from research on social media applications.

Qualitative studies on social media use have reported various instances where ALERT-like pairs of design and experience manifest. Such instances are often represented as ‘distraction’, yet it is important to stress that ALERT is not negative-valence by default but merely directs attention to a target. Typically, the experience becomes negative when ALERT is unwelcome or felt as excessive due to content or frequency. For example, one of the participants in Mannell’s (2019) interview study, Steve, turns off distracting notifications:

*I don’t have control over when people send me the messages or when I get to see them so I try to do things to control that more. So for example, I try not to log in on Facebook on my app and I turned off all the notification settings for my tablet and my phone.* (p. 82)

Avoiding log-in resonates with my earlier analysis on the potentially overwhelming ALERT in CR. Another frame through which ALERT-like feelings are often discussed is ‘dopamine’ and the related discourse. In this (uncritical) discourse, being alerted to notifications—whether related to gaming, social media, or other applications—is considered a ‘dopamine boost’. In another social media study, Conroy *et al.* (2023) interviewed people who experienced their social media use problematic, including Danielle, who describes phone-checking as follows:

*I realized it’s a level of dopamine that gets released when the use of phone (which) brings a sense of achievement ... your dopamine is a very addictive hormone in your body but it may not be for the right reasons* (p. 5)

People like Danielle may thus perceive ‘likes’, ‘replies’, and other social media notifications brining ‘sense of achievement’ but also ‘not for the right reasons’. To what degree this corresponds to ALERT, as I have described it, remains to be explored but the link between ALERT and some of the negative social media experiences seems to be clear. In the study by Vainio *et al.* (2023), people reported various self-limiting strategies for social media use, and these often focused on reducing the effects of features that incite ALERT, such as blinking and bright signal colours that ‘jump’ from the screen when looking at it:

*I had these routines, like putting my phone in another room or if I go to the university, I turn the screen colors off. As I had noticed [problems] in my phone use, I decided not to look at it all during class* (p. 5)

The examples show how researchers have already identified specific design features such as ‘notifications’ contributing to people’s technology use; however, a ‘notification’ is but a single case of design and does not fully represent ALERT as a dual design-phenomenological vitality structure.

My current belief is that ALERT does not play a prevalent role in the experiences of people with clinically significant videogames-related problems. This follows from my axiom that gaming-related problems rarely derive from ‘distraction’ that is associated with ALERT. Nonetheless, as shown earlier, ALERT features can contribute or support larger design structures. I hypothesize that ALERT is experienced as disturbing when associated with high-stress or high-risk content, such as important social interactions. Therefore, it would make sense that negative experiences of ALERT manifest primarily together with tendencies for anxiety in social media use. Following the same logic as earlier, such nosological pattern would be similar to “Anxiety disorder induced by other specified psychoactive substance” (ICD-11; 6C4E.71). In many cases, anxiety being induced by technology use would represent the scenario more accurately than ‘addictive’ behaviour.

## Conclusions

As a partial solution to the long-term challenges of addressing technology and specifically gaming as ‘addictive’ substances—and as psychologically meaningful interactants in general—I have proposed *vitality structures* as a design-phenomenological framework that can help establish construct validity for related phenomena. Vitality structures are not natural kinds to be discovered but pragmatic constructs, which ‘bond’ specific types of design to their phenomenological correspondence. As such, vitality structures are applicable ‘ideals’ and serve pragmatic philosophies of science: they are useful as long as they communicate what is both identifiable and empirically prevalent.

As examples of practice, I have proposed three common vitality structures (CLIMB, FINAL STRECH, ALERT) that are relevant to videogames that treatment-seeking players have reported as sources of their problems. Each vitality structure was shown to serve distinct functions in relation to differently orchestrated videogames (and non-videogames), thus allowing for specific construct-driven hypotheses on the potentially ‘addictive’ nature of such design. Nonetheless, these are hypotheses and both the validity of the identified constructs and their links to treatment-seeking should be investigated in clinical studies. I hope the design-phenomenological framework of vitality structures makes related efforts on construct validation and application easier and, perhaps also, possible.

## Ethics and consent statement

Ethical approval and consent were not required.

## Data Availability

No data are associated with this article.

## Footnotes

^1^ To counter the expected critical remarks on what is presented as monodirectional here (design → human or human →design), I feel obligation to add that all relationships like this are bidirectional *de facto* (design →← human). This does not exclude the possibility of paying special attention to one direction.
