# Vitality structures in ‘addictive’ game design

**DOI:** 10.12688/openreseurope.17177.2

**Published:** 2024-10-14

**Authors:** Veli-Matti Karhulahti

**Affiliations:** 1 Department of Music, Art and Culture Studies, University of Jyväskylä, Jyväskylä, Finland

**Keywords:** behavioural addiction, gambling, gaming, phenomenology, psychiatry, qualitative, technology, theory

## Abstract

For decades, research on technology use and mental health has been based on the assumption that identifiable structures of ‘design’ are psychologically relevant for their users. This has been central especially for the nosological emergence of ‘behavioural addictions’, which currently include two formal diagnoses on technology use: problems related to playing gambling games (gambling disorder) and videogames (gaming disorder). Alas, the research on identifying ‘addictive’ design structures has suffered from major construct validity issues. To make progress over those issues, I draw from the history of ‘vitality affects’ in psychiatry and introduce *vitality structures* as a design-phenomenological framework that can help researchers conceptualise psychologically relevant ‘bonds’ between entities of game design and corresponding player phenomenology. Vitality structures are not natural kinds to be discovered but pragmatic constructs to be used—and useful as long as they communicate what is both identifiable and empirically prevalent. As a demonstration of practice, I propose working conceptualisations of three vitality structures, which surface in videogames played by gaming treatment-seekers. Systematic research programs for identifying relevant vitality structures can lead to construct-valid and replicable design effects.

## Introduction

My goal here is to contribute to the interdisciplinary research efforts on what the current psychiatric discourse refers to as ‘addictive behaviours’. I focus on one such behaviour—gaming, i.e. videogame play—due to its status as one of the two diagnosable mental disorders in the *International Classification of Diseases* (ICD-11) in this category. To better conceptualise those ‘addictive’ videogame mechanisms that the related clinical research programs imply, I propose a dual design-phenomenological framework based on *vitality structures*: dimensional construct ‘bonds’ between identified units of experience and technology. I hope vitality structures can serve as a step toward more pragmatic and construct-valid approaches to explaining how people interact with contemporary and future technologies. To demonstrate that potential in practice, I propose three distinct vitality structures as starting points to be further refined and empirically tested in the future. 

The first sections introduce terminological clarifications, review relevant practical and theoretical literature, and outline the vitality structure construct as a new unit of analysis. The second half proceeds with analytical and theoretical scrutiny by identifying exemplary vitality structures as well as elaborating on the ontology of the construct.

### On Terminology

I keep using marks around ‘addictive’ due to the still open research question about whether framing technology ‘addictive’ makes scientific sense (e.g., Billieux *et al.*, 2017; Ferguson & Colwell, 2020; Kardefelt-Winther, 2017; Sutton, 2020). Most experts—and especially non-experts—would agree that contemporary products that utilise the internet, mobile devices, and the computer are efficient at capturing our attention continuously. From this viewpoint, it may be reasoned to call such technology ‘addictive’ just like good books, television series, and physical exercise can be ‘addictive’ in many ways (see Bell *et al.*, 2015; Billieux *et al.*, 2015; Brus, 2013; Calvo *et al.*, 2018). Less controversial synonyms could be applied here too, such as ‘engaging’, yet because the goal is to contribute to the clinical discourse, using ‘addictive’ from a healthy critical distance feels justified. Moreover, we should remind ourselves that no substance, such as alcohol, is ever exclusively one thing (‘addictive’) but always many things simultaneously (tasty, stimulating, etc.).

In psychiatry, too, there has been increasing doubt toward the term ‘addiction’ and related terms nosologically (see Barry *et al.*, 2014; Botticelli & Koh, 2016; Broyles *et al.*, 2014; Matthews *et al.*, 2017). In the most recent *Diagnostic and Statistical Manual of Mental Disorders* (DSM-5-TR; 2022) the “more neutral term *substance use disorder* is used to describe the wide range of the disorder” (p. 543), covering drugs as well as gambling. However, the term ‘addictive’ remains in their chapter title and also the ICD-11 has a kept the word ‘addictive’ as a topic label for “disorders due to addictive behaviours” (for a recent perspective to classificatory challenges, see West *et al.*, 2023). Notably, this category is the one where also videogame-related health problems have their own formal diagnosis as *gaming disorder*.

Using the term ‘addictive’ with reference to videogame design and play can be tautological, nonetheless. For a design to be classified as such, it is necessary to identify the ‘addictive mechanism’, i.e. how a design contributes to a type of behaviour; while the ‘addictive’ nature of the behaviour remains defined by the behaviour itself. I spell out the two key problems.

First, a construct-valid search for ‘addictive’ designs entails someone being ‘addicted’ to certain designs—the existence of such design depends on the existence of a corresponding experience. Being able to say “ *that* gaming experience is/isn’t addicted” is a premise for outlining any (non) ‘addictive’ design, and we need to agree upon what that experience is to identify relevant designs. For example, it is unclear whether ‘addictive behaviour’ should equally apply to someone being distracted by checking their smartphone regularly (e.g., Conroy *et al.*, 2023; Vainio *et al.*, 2023), and another person playing a massive multiplayer online game for decades (e.g., Bleckmann & Jukschat, 2015; Domahidi & Quandt, 2015). Should ‘distractive design’ be separated from ‘addictive design’? We have no space for answering such questions here, but being aware of them will help us move forward.

Second, delineating an ‘addictive’ design *per se* as, say, alcohol or nicotine remains a holy grail in the field; also in the sense that we do not know if it exists. As Panova & Carbonell (2018) note, “smartphone addiction” is the same as “drinking glass addiction” would be for alcoholics—identifying specific ‘addictive’ elements *in* design remains a challenge both methodologically and theoretically. For instance, one could propose the ‘red dot’ appearing in many smartphone applications as one type of ‘addictive design’ but such delineation would hardly be sufficient. The ‘red dot’ would need to be contextualised in the larger design framework of notifications (i.e., if and how it is different from other notifications) and applications where it occurs. When sufficiently contextualised, the connection between that construct and a user’s experiential engagement with it should be demonstrated. This can produce a hypothesis of the underlying clinical mechanism(s) that explain ‘addictive’ interaction with it. Taken together, the present scenario can be described as one of *epistemic iteration* ( Chang, 2004) where successive stages of scientific knowledge are being built on imperfect preceding ones. Chang’s example of scratched glasses is helpful here:

*Without wearing my glasses, I cannot focus very well on small or faint things. Therefore if I pick up my glasses to examine them, I am unable to see the fine scratches and smudges on them. But if I put on those same glasses and look at myself in the mirror, I can see the details of the lenses quite well. In short, my glasses can show me their own defects.* (p. 230)

Although we do not have a good understanding of ‘addicted’ gaming yet nor do we even agree whether ‘addicted’ behaviour is the right framing, we can use the state of art as a stepping stone without commitment. To make progress, we need to anchor the present gold standard of ‘addictive behaviour’ on the best available data. This will serve as an auxiliary hypotheses, which can later be corrected or rejected in the light of better data. Currently, the best data come from actual treatment-seekers who have problems with gaming.

Having addressed the ‘addicted’ experience, I should further highlight my use of the term *phenomenology* in the present design-phenomenological framework. Although phenomenology remains a contested conceptual (mine)field, the clarification here should be straightforward due to my relatively faithful application of the term in “its original sense” (with a grain of irony; see Smith, 2018; van Manen *et al.*, 2016; van Manen, 2017). In the next pages, as I introduce and investigate vitality structures, the frequent references to phenomenology are very phenomenological indeed: the vitality in question follows a relatively conventional (but not a fundamentalist) reduction of experiences into as clean as possible, dimensional analytical particles, which are conceptually independent of the rest of one’s past, present, and future (for a conceptual rview, see Zahavi, 2019).

Although my use of the words ‘experience’ and ‘phenomenology’ will be more synonymous than not, a distinction between the two should be made. I see the former generally referring to a wider unit, which takes into consideration one’s past, present, and future through various contextual and personal aspects, whereas the latter more narrowly captures the very shape of the lived moment without such context. That said, my forthcoming use of both words will always assume an empirical co-dependency between the two, especially when it comes to interpreting clinical data. Every human datapoint surfaces from life context, which makes pure phenomenological description flawed in practice; simultaneously, phenomenology remains the best available tool for producing the kind of pragmatic knowledge that explains human behaviour in the life context. In crafting psychologically relevant constructs, I am in concert with Husserl (1900/1973), Heidegger (1927/1996), and others who argue that an ontology for a science derives from phenomenology. Accordingly, the identification of phenomenological vitality in gaming experiences is a necessary step toward both pragmatic and theoretical knowledge of constructs, which can better explain technology use.

### On Design

Next, I address the problems that have limited previous research on design structures. Identifying such structures as constructs that can be re-identified across different videogames (and non-videogames) is a challenge both methodologically and theoretically. I find the research on the design of gambling games as the most fruitful starting point for two reasons: ‘addictive behaviours’ related to gambling are qualitatively well documented over several decades (e.g. Blaszczynski & Nower, 2002; Livingstone, 2005; see also Calado & Griffiths, 2016), and the designs that engage problematic gamblers have been studied longer than videogames have ever existed.

The designers of gambling games are known to have identified several mechanisms with a high probability of contributing to people’s gambling behaviours. It was B.F. Skinner himself who suggested no later than in the 1950s that conditioning had already been efficiently integrated to gambling design, as the “efficacy of such schedules in generating high rates has long been known to the proprietors of gambling establishments” ( 1953/1965, p. 104). I consider Skinner’s insights valuable because they infer directly from (behaviourist) theory. For example, Skinner clearly described what is nowadays known as ‘near-miss’ as follows:

*Gambling devices make an effective use of conditioned reinforcers which are set up by pairing certain stimuli with the economic reinforcers -- “almost hitting the jack pot” increases the probability that the individual will play the machine* (p. 397)

For good reasons, we thus see in-depth investigations of near-miss already in the 1980s ( Reid, 1986). In an influential recent study, Clark and colleagues (2009) found players who obtained near-misses to experience their chances of winning higher and expressing greater desire to continue playing, compared to those having regular losses. Dixon and colleagues (2013), in turn, found near-misses being associated with large skin conductance responses (which typically occur in winning) and short post-reinforcement pauses (which typically occur in losing). Although the qualitative structures of near-miss experience remain largely unmapped, the above and similar findings ( Barton *et al.*, 2017) can be interpreted as players affectively experiencing near-misses akin to winning, but immediately after the affective response update the experience into a distinct type of unpleasant loss.

Alas, not all designs of near-miss seem have similar effects. For instance, in slot machines, the effects appear to manifest only if the last reel(s) produces near-miss ( Dixon *et al.*, 2013), and an increase in the desire to play more occurs only when the near-miss is due to the player’s own interactive agency ( Clark *et al.*, 2009). To further complicate such designs, the frequences of near-miss are also likely to play a role in experiencing them—as when encountering many near-misses in short period of time desensitises the player’s response to them ( Kassinove & Schare, 2001). Consistent with such caveats, a systematic review of different types of near-miss found them to be associated with

*increasing one’s bet, decreasing one’s bet, or having no effect, each in a different study, making it difficult to determine whether near misses are capable of influencing per-play betting behaviour* -- *without more stringent control and investigation of gambling phenomenology, it is difficult to conclude how exactly near miss events are affecting the player* ( Barton *et al.*, 2017, pp. 1254–1255)

Despite the general consensus that near-misses affect most players in some meaningful ways, up to the present day, it remains unclear how near-misses manifest phenomenologically and whether such manifestations have generalisable effects on gambling behaviour. A recent failed replication review ( Pisklak *et al.*, 2020) persuasively suggests that, for half a century, “near-miss research may have been misguided from the start” (p. 629), and perhaps the psycho-phenomenological construct of engagement due to near-miss design manifests only in a yet-unidentified limited context and/or “as initial machine selection or amount bet” (p. 627; notice that this was Skinner’s original proposal!). In other words, the construct(s) do not seem to be clearly enough understood to sufficiently inform and organise replicable experiments. A further understanding of such constructs would need to bring attention to the dimensional bonds between designs and experiences, which have hitherto lacked conceptual identity.

Similarly, already in the 1970s, Langer (1975) proposed ‘illusion of control’ as a construct that represents inappropriately high expectancy of personal success in gambling and other chance situations when agency is involved. Over the decades, the construct has accumulated large amounts of research and there seems be a consensus that people prefer exerting agency (e.g., by choosing their own lottery numbers) even when they admit the outcome to depend on randomness (e.g., Clark & Wohl, 2022). For example, when players are provided with interactive agency, letting them win more frequently at first may increase their belief in winning ( Eben *et al.*, 2023a; Eben *et al.*, 2023b), as may do experiencing near-misses ( Clark *et al.*, 2009).

However, illusion of control effects, too, are difficult to match with players’ experiences thereof. From the very beginning, Langer herself (1975) was open to the idea that players prefer control because they find choosing “meaningful to them -- rather than an increase in confidence in winning” (p. 323). Failed replications support such non-illusory, alternative player experiences. For instance, an impressive replication of 17 experiments consistently found that choosing did not make people feel more likely to achieve preferable outcomes when the options were functionally identical ( Klusowski *et al.*, 2021; see Wong & Austin, 2008). This is not surprising from a phenomenological viewpoint, as many gamblers who identify their play as ‘addicted’ do not even care about winning—as says Mollie, one of Schull’s (2012) addict-identifying interviewees: “The thing people never understand is that *I’m not playing to win*” (p. 2). Even if players, under an illusion of control, believe more in winning, why would that matter if they do not care about winning in the first place?

A recurring issue that hinders the research on gambling design is the lacking attention to the duality of such constructs—variations in design *and* phenomenological response. In practice, this means that designs like near-miss surface in numerous different forms, and players experience such designs in numerous different ways. When some players do likely experience feelings in the range of illusory control via diverse design interactions, others may feel and act similarly (and differently), for example, to simply enjoy a sense of autonomy and independence (e.g., Reiss, 2012; Ryan & Deci, 2017; for alternative psychological responses to such designs, see also Loftus & Loftus, 1983). Instead of focusing on isolated designs and effects, the theoretical lens needs to move to carefully selected bond networks where the spectra of designs meet the spectra of experiences.

Things get even more complex in videogames, however. Whereas all gambling is based on relatively simple design mechanisms involving random rewards—and gambling products are highly accessible in general due to their economic vector (see Karhulahti & Grabarczyk, 2021)—the most played videogames like *League of Legends* and *World of Warcraft* are extremely multifaceted and highly dynamic design products. Despite the fact that constructs like near-miss and illusion of control might be possible to identify as small particles of such games, evidencing their effects in such context remains a challenge. A possible methodological alternative would be to deconstruct entire videogame designs or their segments to identify central patterns (e.g., Järvinen, 2008; Lankoski & Björk, 2015; Sicart, 2008); however, creating a system for classifying different designs remains a meta-challenge that keeps slowing down progress in this regard.

A decade ago, I made a still-relevant distinction between ‘augontological’ and ‘typontological’ approaches to the analysis of videogame structures ( Karhulahti, 2015). In the former, the idea is to map out all prevalent or relevant particles of games, such as design patterns and elements. For example, both Game Ontology ( Zagal *et al.*, 2005) and Gameplay Design Patterns ( Björk & Holopainen, 2005) collections include an entry called Scores, which represents a central element of design that can manifest in various ways across different videogames (and non-videogames). There is literally an infinite number of concepts like Scores, and such collections can thus be expanded infinitely. There are currently 623 gameplay design patterns officially listed ( http://virt10.itu.chalmers.se/index.php/Main_Page). Some of these patterns (e.g., Grinding) are classified as ‘dark’ ones ( Gray *et al.*, 2024; Lewis, 2014; Zagal *et al.*, 2013), as they possibly contribute to negative gaming experiences (e.g., by artificially increasing expected play time).

Typontological approaches, in turn, do not aim at covering all elements but rather the dimensions by which such elements are built. For instance, in the typology by Elverdam & Aarseth (2007), there are no elements such as Scores but rather a dimension of Struggle that is divided into Challenges and Goals, and the latter further split into Explicit and Implicit goals. A score design could thus be an instance of an Explicit goal. Again, it would be possible to further expand a dimension like Struggle or Goals with an infinite number of additional subdimensions. As different research questions entail different degrees of depth, choosing optimal accuracy remains a pragmatic matter.

The clinical research domain has addressed videogame design as well. For instance, King & colleagues (2010) have proposed a list of fundamental ‘psycho-structural’ elements of videogames to be applied in the study of mental health. The list consists of five main categories (social, manipulation, narrative, reward, presentation) that were largely self-selected with the guidance of gambling research (see Wood *et al.*, 2004). Despite their intended clinical application, such lists do not derive from actual cases of health problems that manifest with specific videogames. In consequence, they do not have the depth of augontological and typontological systems, but also lack the clinical dimension. On the other hand, outside the domain of gaming, clinical paradigms of behaviour change ( Michie *et al.*, 2011) are also actively mapping out the techniques that operate like videogame elements ( Michie & Johnston, 2012). Although related, the links between these everyday ‘active ingredients’ and videogame design remain distant to such extent that they need to be charted elsewhere.

Finally and recently, Flayelle and colleagues (2023) have approached technology analysis from a point of view that connects specific psychological mechanisms to potentially problematic design features. For example, affective mechanisms include processes such as emotion dysregulation, which in interaction with design can contribute to impaired control. Meanwhile, Meier & Reinecke (2020) have suggested a distinction between channel and communication centred approaches to health research on social media: the former focusing on design (application, feature, etc.) and the latter on how the design is interacted with (configuration, directionality, etc.). Although neither of the above studies investigate specific psychology-design combinations but review previous literature, such dual approaches are relevant to the present work and I elaborate on these links later.

### On theory

Merton (1949) famously coined the attempts to refine precise everyday observations into coherent holistic systems as “theories of the middle range”. The intersection between the human user experience and more general systems (and practices) of design forms an optimal field for middle-range theories—in concert with the previously argued need to draw stronger links between specific designs and experiences. I review such theories in this section, with a caveat that some of them better serve as ‘conceptual frameworks’ that do not pursue explicit theoretical utility, such as explanatory or predictive power. Most of this work comes from the field of human-computer interaction, which itself represents an intersection of technologies and their use.

In a seminal effort to conceptualise human-computer interaction, Dourish (2001) looks at a range of interactive patterns through the concept of *embodied interaction*. One of the related key arguments is that, in the context of computer use, effective designs “exploit our familiarity and facility with the everyday world—whether it is a world of social interaction or physical artifacts” (p. 17). This means that gaming players construct meaning for such interactions in the same way as they do for other daily interactions (see also Hassenzahl, 2010). Ultimately, embodiment is the “property of our engagement with the world that allows us to make it meaningful” ( Dourish, 2001, p. 126), and this very embodiment can also help to bridge player phenomenology and videogame design (e.g., Vahlo, 2017; Vahlo, 2018). To a large extent, embodied subjectivity determines how design interactions are experienced and gain meaning.

Embodied and enactive approaches to cognition (e.g., Varela, 1972/1980; Varela *et al.*, 1991/2017) are not limited to effective interaction but also concern perception (e.g., Johnson, 2007; Noë, 2004; Noë, 2012). In the context of gaming, this means that players feel, hear, and see the designs of their videogames differently depending on their subjective life histories and bodies—an insight that phenomenologists made a long time ago (e.g., Gadamer, 1960/2006; Merleau-Ponty, 1956/2013) and continue to discuss in more modern technological contexts (e.g. Hansen, 2004; Verbeek, 2008) and ‘postphenomenology’ in particular ( Ihde, 2019). An important lineage dovetailing these premises is the ecological approach and its *affordances*, which has also found its way to the analysis of technology design (for a short history, see Suoranta, 2024).

The theory of affordances evolved over decades in the work by Gibson ( 1950, onwards), eventually being defined as “something that refers to both the environment and the animal -- properties of things *taken with reference to an observer* but not properties of the *experiences of the observer*” ( 1977/2014, pp. 119, 129). A specific design of a videogame affords different things to different players; those affordances should not be confused with the players’ differing experiences, but they rather exist ‘between’ the design and the player. The notion of affordance has been applied to communication, design, and gaming research through various interpretations (e.g., Evans *et al.*, 2017; Linderoth, 2013a; O’Neill, 2008).

Zhang (2008) has proposed ‘motivational affordances’ as a specific case of the Gibsonian affordance for design research, namely, the “properties of an object that determine whether and how it can support one’s motivational needs” (p. 145). The idea is put forward under the theoretical assumption that humans follow basic motivational needs, and design products such as videogames operate largely by satisfying such needs for their players (e.g., Hassenzahl, 2010; Rigby & Ryan, 2011; Ryan *et al.*, 2006). Deterding (2011; 2014) takes the proposal one step further by adding that each affordance in design functions in the dynamic context of play: “the transfer of a design element [into] another usage context likely does not necessarily lead to the same motivational affordances -- we have to conceptualise them as necessarily *situated*” ( 2011, p. 3; see also Lewis, 2014). A new theoretical question thus arises regarding relevant variables in each context and situation, which should be considered for a design to properly serve its intended purpose, or ‘desiderata’ ( Nelson & Stolterman, 2012).

Next to the above, it is also worth addressing an alternative embodiment framework that Lim and colleagues (2007) have labelled the ‘interaction gestalt’. Repeating what I have said earlier, the authors note that existing approaches “have a large gap between *use qualities* and *artifact properties* which designers need to bridge” (p. 246). Drawing from the somaesthetic branch of embodiment theory (see Shusterman, 2008), they propose interaction gestalt as the shape of a particular interaction, which is possible to describe by a set of diverse attributes:

[one] *has to separate the user experience and the artifact properties from the interaction itself -- interaction is an abstract entity that* emerges *between the other two entities. However, even if the interaction is an abstract entity, we see that it can be directly designed. A designer can, for instance, decide to make the interaction* slow *or* fast *,* static *or* dynamic ( Lim *et al.*, 2007, p. 240)

The authors suggest time, space, and information as the main factors, on which most interactions can be descriptively mapped. This framework is, to my knowledge, the first one to take construct dimensionality seriously and comes closest to the upcoming vitality structures (see also Swink, 2008; note overlap with the MDA framework in Hunicke *et al.*, 2004).

Although I am not aware of studies that would have attempted to model interaction gestalts empirically, related efforts have been exerted elsewhere. For instance, collative variable pairs such as ambiguity-clarity, complexity-simplicity, expectedness-surprise, and familiarity-novelty have been modelled to explain and predict human responses to artworks in empirical aesthetics ( Marin, 2022). The same challenges as noted earlier—sufficiently acknowledging construct duality and addressing contextual, situational, and subjective diversity—hinder replicability as well as complicate the identification of operable constructs in that tradition (ibid., p. 412). Following these persistent challenges, it is time take the initial steps toward the framework of vitality structures, which may help solving some of these issues.

### Vitality Structures

My philosophy of science is pluralist and pragmatic ( Chang, 2023), that is, I believe multiple competing programs of knowledge can exist at the same time as long as they all contribute to operationally coherent practices. It can be useful to keep running better experiments for expected design effects and building better (or bigger) classification systems of design—while at the same time, none of these efforts alone or separately are sufficient to capture what is needed to understand ‘addictive’ videogame design, whatever that comes to mean. My diagnosis of the issue is that related discussion often takes the ‘design’ as a starting point—i.e. a certain type of common design element—but fails to acknowledge the phenomenological (not merely behavioural) counterpart that connects to it. What we should care most is the human experience: not asking “what does a *design do*” (design → human) but rather “how did a person end up *feeling that*” (design ← human)? I aim to follow the latter epistemic sequence below. ^1^

My proposed design-phenomenological framework is motivated by the idea of ‘vitality affects’, as coined by developmental psychiatrist Daniel Stern in the early 1980s. The concept has developed over four decades ( Koppe *et al.*, 2008) and there is no one ‘true’ definition or interpretation of it. Koppe *et al.* (2008) offer a conceptual review of the concept’s history and summarise the initial form as follows:

*Vitality affects are connected to vital life processes such as breathing, becoming hungry, falling asleep, waking up, etc., to which the term ‘‘vitality’’ refers. They are, however, also present in principle in the carrying out of any kind of goal-directed mental activity, for example a thought progression, an interaction, or a dialogue. Stern exemplifies [these] vitality affects via transitory qualities: they are something ‘‘surging,’’ ‘‘fading away,’’ ‘‘exploding,’’ etc* ( Koppe *et al.*, 2008, pp. 170–71).

The authors suggest that some of the examples, such as vital body rhythms (breathing), should not be considered features of vitality affects because they tend to confuse the concept with general feeling(s) of a body. Instead of being associated with natural bodily functions, the conceptual focus should be on amodal, relationally felt aspects such as changes in direction, speed or intensity over time. I believe this is a useful criticism, so let us keep that in mind.

Two years before passing away, Stern (2010a) published a book titled *Forms of Vitality* where the conceptual target moves from affects to forms (of vitality). Forms of vitality are more clearly defined in relation to dynamic events. Stern names five elements of which these dynamic forms consist: movement, force, space, diction/intention, and time. The reference to physics is not a coincidence (he mentions Einstein’s description of ‘thought’ as inspiration). I cite Stern at length:

*I want to suggest that there is a special way that we experience dynamic events. Now when I say dynamic events, what I mean is events that unfold over time, that have a force apparently within them or behind them, that are going somewhere – these events, and that they seem to be pulled towards some goal. The important thing is the event seems to consist of some kind of movement, and it takes time, and it also occurs in some kind of space, even if it’s mental space... I am going to call them Forms of Vitality* ( Stern, 2010b, p. 88)

This is where I pause and hope you have a tentative picture of what I have in mind when using the term ‘vitality’. To be clear, I am not interested in debating about the ‘true’ nature of vitality as expressed by Stern or someone else; rather, my goal is to communicate a certain type of vitality, which I learn from Stern’s description: a feeling of (not random but) directional/intentional movement in mental space that has some force, and this movement naturally manifests over time as we experience it. These are dynamic qualia, which are present almost every moment in our lives as we are awake. For example, drinking from my coffee cup involves numerous ‘forms of vitality’ from the specific “touch of the cup” (warmth that starts with short contact and expands) to the feeling of “coffee running out of the cup” (‘lessening’ in a sense that there is less and less of it as I drink), and to the feeling of “last sip” when I empty the cup (void after the fact). In all these examples, different types of movement with force occur in space through time and they have certain ‘intention’—not in a sense that it would be *my* intention or action, but the force has its own intention as ‘directed’ or ‘determined’ course, as when the coffee “runs out.”

**Figure 7.**
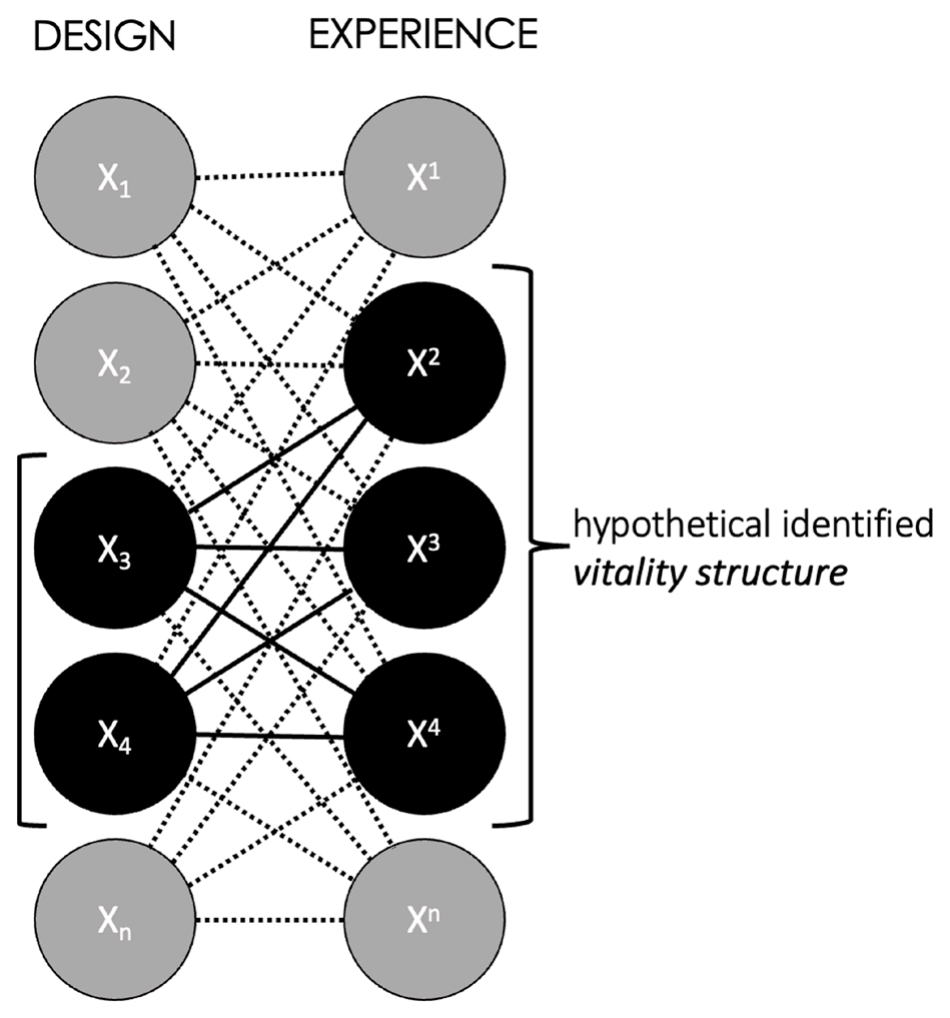
A network of bonds between variations of design and experience. When designs that are external to the vitality structure (X _1_, X _2_, X _n_) bond with experiences that are internal to it (X ^2^, X ^3^, X ^4^) and vice versa (X _3_, X _4_ bonding with X ^1^, X ^n^), these may occur e.g., in case of self-created challenges, transgressive play, and gaming experiences influenced by sociocultural dynamics (see e.g., Iversen, 2012; Nakamura & Wirman, 2005; Zhu, 2015). In brief, a player not meeting the designed ‘implied player’ position ( Aarseth, 2014) is often sufficient to short-circuit the identified vitality structure bond. Author’s own graph, no further permission needed.

From the above, I derive *vitality structures* as ‘bonds’ between technology and human experience—theoretically warranted constructs, the long-term validation of which is a matter of both design and phenomenological experiments. I stress that vitality structures are not only features of design, nor merely something experienced. They are constructs that bond *specific* designs with *specific* experiences. Although one may use vitality structures in reference to either the design-side or the phenomenology-side of the construct, it is important that neither can exist without the other (see Figure 6 in later ontological discussion). The ‘structures’ of vitality structures highlight the dual nature of the design-phenomenological framework: it is a structured experience, as specific videogame (and non-videogame) designs can be built to provoke the experience, but the design alone is not a vitality structure until we identify the corresponding phenomenology (see Figure 7 in later ontological discussion). It is possible to interact with the design-side of an identified vitality structure *without enacting the corresponding phenomenology*, and it is also possible to have the corresponding phenomenology in everyday life *without such design interaction*. It is the bond between a design and its phenomenological correspondence that delineates vitality structures as dualities—after a specific bond has been identified, its sides can be addressed in isolation.

**Figure 1.**
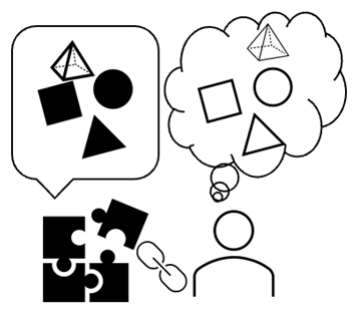
A videogame involves vitality structures by design, and players attune to such vitality structures by playing. *Author’s own graph, no further permission needed*.

I want to avoid overly metaphors, but one will aid us to better grasp what is being discussed. For Stern, vitality surfaced at first in the infant-mother relationship (see Ammaniti & Ferrari, 2013; Modell, 2018) where the two have no shared language yet, but they can feel or sense what the other is feeling or sensing by perceiving corresponding vitality forms and (re)enacting them. Stern calls this *affect attunement*. By reacting to and with gestures, sounds, and other means, the amodal vitality forms can be conveyed from one human to another. My intention is not to anthropomorphise technology, but I believe it helps to think about the human-technology relationship in a similar way: non-speaking applications like videogames conveying to their players ‘vitalities’ that have no corresponding words but ‘forms’. Stern (2010a) would have agreed with this metaphor, as his very idea was that “vitality forms are readily transferable between art forms” (p. 79). When people play videogames, a type of affect attunement takes place: players attune to specific forms of affective engagement that someone designed ( Figure 1). This is fully consistent with playcentric videogame design, which starts from the designer identifying a target experience to be conveyed for a target player group ( Fullerton, 2004; see also Nelson & Stolterman, 2012, pp. 159–171).

The attunement of vitality structures also reminds us of John Dewey’s (1934) classic theory of artworks as ‘experiences’ that manifest when people interact with artefacts that bear aesthetic qualities. Although a product of art has an increased probability to trigger a (specific) aesthetic experience in a person, aesthetic experiences may occur also as the “zest of the spectator in poking the wood burning on the heart and in watching the darting flames and crumbling coals” (p. 3). Likewise, a certain ‘addictive design’ may be prevalent in videogames, but its corresponding phenomenology can also be unintentionally experienced elsewhere.

What I have so far referred to as ‘corresponding phenomenology’ is a very empirical matter: to what degree people’s experiences of an identified vitality structure actually correspond with a design becomes a research question to be investigated and replicated. This is comparable, for instance, to the ongoing work on prelinguistic structures such as ‘image schemas’, which Johnson (2007) describes as

*recurring pattern of organism-environment interactions -- ask yourself what are the most fundamental structures of perception, object manipulation, and bodily movement, given that human bodies share several quite specific sensorimotor capacities. [Because] of our particular bodily makeup, we project right and left, front and back, near and far* (p. 75)

Other common image schemas include ‘containment’ (the idea that something is inside or outside) and ‘blockage of movement’ (dynamic counterforce). The latter in particular closely resemble Stern’s vitality forms, and serve as empirical and theoretical extensions thereof. Unlike vitality forms, image schemas—and their underlying ‘primary metaphors’ such as “more is up / less is down”—have been subject to numerous empirical studies, especially in relation to aural perception and musical forces ( Johnson, 2007, p. 168–169; for a unique bodily experiment, see Tuuri & Pirhonen, 2013). This empirical work is generally linked to crossmodal correspondence, such as the systematically replicating kiki/bouba effect: people across cultures visualise the ‘kiki’ sound as sharp the ‘bouba’ sound as blunt ( Ćwiek *et al.*, 2022). The same prelinguistic forms appear to be connected aurally and visually by other animals too, including baby chicken ( Loconsole *et al.*, 2024).

Taken together, there are good reasons to believe that specific designs in videogames and other technological products can evoke specific phenomenological responses across individuals, and it is possible to identify such bonds as empirically testable vitality structures. I postpone deeper ontological discussion of vitality structures to the end of this article, which will be easier to follow after hands-on investigation of vitality structures in practice. In the upcoming pages, I thus set out three hypothetical vitality structures for scrutiny and testing.

## Methods

A recap is in order. The first challenge of studying ‘addictive designs’ is to know what ‘addictive behaviour’ related to a design is; only with a good auxiliary hypothesis regarding a certain design being involved in such experiences can we identify relevant designs. The second challenge is to effectively delineate a design and its corresponding phenomenology—constituting the underlying clinical mechanism(s). Merely naming a common design feature or experience is not useful, but they need to be identified with sufficient accuracy and the links between them properly outlined. In this way, we can move toward hypotheses of selected design-phenomenological pairs, to be tested for their ‘addictive’ nature across different contexts, situations, and people.

My basis for the present work shall be a list of videogames played by actual treatment-seekers in Finland. In our earlier research ( Karhulahti *et al.*, 2023a), we studied the reasons of gaming-related treatment-seeking with 110 participants who had applied for local clinical services. The participants named 26 unique videogame titles more than once as sources of their problems, and the three upcoming vitality structures come from these titles. Importantly, we currently lack in-depth data regarding *how* the participants experienced specific titles as problematic. Therefore, the present vitality structures may not fully correspond with the problems experienced by those individuals.

As has become clear, my proposed solution is an approach where the delineation of a construct flows primarily from human to design, and is iterated in a design-phenomenological framework. To access the phenomenological domain and be able to identify potentially relevant ‘addictive’ designs, I refer to my own experiences as a player of three videogames, which multiple treatment-seekers named as sources of their problems. I do not consider my experiences ‘addicted’ but highly ‘intensive’ nonetheless. Arguably, this makes me imperfectly but still well positioned to identify potential ‘addictive’ designs in these titles.

In the related fields of games research, human-computer interaction, psychiatry, and psychology, ethnographic and qualitative methods are typically applied to understand the player experience (e.g., Desjarlais, 2011; Macey *et al.*, 2024; Markham, 2013; Rapp, 2021; Sudnow, 1983). The present theoretical work does not use such methods but retrospectively reflects on my own ‘organic’ player experiences in the past. Such reflections of the past cannot and should not be compared to established ethnographic or qualitative traditions, but a careful application of memory can hold phenomenological value in its own right.

In an important methodological account of ‘remembering research’, Keightley (2010) convincingly argues that “transparency and scrutiny are crucial if there is to be a broader acceptance of the empirical value of memory studies in the social sciences and beyond” (p. 67). I agree with Keightley and, following her advice, aim at transparent recalling of my past experiences without claiming to have produced such information as an outcome of systematic note-taking, planning, and analysis. Instead of such steps, I disclose relevant details about the chosen titles below and, after that, elaborate on the experience recall of the examples.

***League of Legends (LoL)*** is a team esports videogame that represents the so-called MOBA (multiplayer online battle arena) genre. Matches are played in 5-player teams and last approximately 25–30 minutes. I played LoL competitively for years (approximately 3,000 hours), details of which are documented elsewhere ( Karhulahti, 2020a). After quitting in 2018, I have followed the game’s evolution from a distance. According to the game’s open API, I have nonetheless played 136 hours in the last six years. Recently, I have also played the almost identical mobile version *Wilf Rift* (WR) with some competitive motivation, yet with more modest efforts (approximately 200 hours overall).

***Clash Royale (CR)*** is a competitive mobile card game. Matches are played 1v1 in a tower defence setting, and they last 2–5 minutes. Because success in CR is partly based on card strength that can be maximised only by continuous microtransactions, my competitive motivation is limited (I am against pay-to-win designs in principle). I have played CR since 2018 as a daily pleasure. According to the open API, my lifetime play investment is 781 hours. The game is designed to encourage frequent daily log-ins, so I play a few times per day (about 20 minutes in total).

***Soulsbornes (SB)*** are single-player action roleplaying storygames, which also have optional multiplayer modes. The series is known for its high difficulty and includes six independent instalments, which are mechanically almost identical: *Demon’s Souls* (2009), *Dark Souls* (2011), *Dark Souls 2* (2014), *Dark Souls 3* (2016), *Bloodborne* (2015), and *Elden Ring* (2022). I completed all titles once and each took about 100 hours of playtime, thus I have spent about 600 hours on Soulsbornes. I did not engage in their competitive multiplayer modes, albeit some of them enforce occasional engagement with other players.

I utilise these title-specific histories of engagement to propose three vitality structures that appear frequently in one or more of these videogames. The conceptualisations of these vitality structures and their phenomenological relevance for intensive gaming have evolved in my gaming experiences over the past decade. Since 2010, I have drawn and written unstructured memos whenever subjectively interesting research insights have spawned from free time gaming. In general, those notes do not follow a specific topic or perspective, but rather represent exploratory ideas, theoretical sketches, and hypotheses. In the course of such ‘continuous introspection’, some of the ideas gradually found shape as vitality structures. For instance, in previous work I have discussed the experience of climbing at length ( Karhulahti, 2020a) but was not able to conceptualise and contextualise it as a vitality structure. This can be partially characterised through an element of phenomenological reduction in the clinical tradition ( Stanghellini *et al.*, 2019), but it would not be correct to describe the process as such. Rather, after the recurring experiences over years, a form of reduction was undertaken in the writing phase of this article by re-returning to the selected experiences to conceptualise them in sharp dimensional terms (see Varela & Shear, 1999).

In contemporary philosophy and that of technology in particular, ‘conceptual engineering’ has manifested as a trending methodological frame for identifying concepts and their problems (e.g., Floridi, 2011). I did not explicitly follow any specific protocol for ‘engineering’ or ‘reengineering’ vitality structures, yet my processes of conceptual clarification and specification do not differ meaningfully from such approaches. I revised visual drafts, maps of relevant terms, and alternative definitions to improve the concept of vitality structures until a satisfactory dimensional degree was reached. A critical aspect in the process was an organised scrutiny of the concept against the existing literature: feedback from colleagues and reviewers led to further revisions, of which the most recent version rests here. The descriptions of the identified vitality structures are not meant to be final but starting points for refinement and formalisation. Reassessing them against and with future empirical data is likely to introduce more clarifications, especially when operationalised quantitatively. Merton (1987) would call this identified gap in my current knowledge *specified ignorance*, which is a necessary feature of all sustainable scientific progress. It remains to be seen to what degree currently popular forms of data, such as online surveys, are capable of measuring vitality structures and what other means of measurement become possible.

I do not have evidence nor do I claim that the chosen vitality structures are the most central in terms of ‘addictiveness’. Rather, I have chosen them because they represent a good variety of differently relevant vitality structures and serve as helpful illustrations.

## Analysis

Vitality structures are not natural kinds. By this I mean that each vitality structure is an abstract representation of affective mental movement: what people feel or sense when interacting with a design—or from the other point of view, mental movement that was designed to be felt or sensed. Two feelings can never be exactly the same, and there is usually some overlap with two different feelings. This does not mean that vitality structures cannot be studied empirically or they have no empirical basis; quite the contrary. A good vitality structure should empirically resonate with the experiences of multiple people, and this correspondence should be validated by empirical means. The present autographic reflections of the past are one such limited means, and a beginning for further development.

For the vitality structures to take dimensional shape, I craft these constructs through macro, meso, and micro *chronotopes*. By chronotope I refer to experienced spacetime; both the ‘size’ and ‘length’ of the experience, as further explained later. Naturally, the three chronotopes are not clearcut but represent a continuum. A macro-chronotopic vitality structure is felt via ‘expansive’, ‘prolonged’, and ‘slow’ mental movement. A meso-chronotopic vitality structure is felt to be ‘faster’ and more ‘intense’, and a micro-chronotopic one is ‘immediate’ and ‘sharp’ as if experienced in the very moment. Finally, a vitality structure can manifest as one, two, or all three chronotopes: a micro-chronotopic structure may sometimes be bent into meso- and macro-chronotopes, and vice versa. I understand that my words cannot fully communicate the intended qualia. I hope the upcoming examples do better.

The chronotope involves both spatial and temporal description, but each can be further expanded. I will thus add two meta-dimensions across which vitality structures can extend: *meta-spatial* and *meta*- *temporal*. By meta-spatial I mean whether the vitality structure is experienced ‘close’ to the player’s self or farther away, as through an avatar or object. Examples will clarify this soon. By meta-temporal I mean whether the feeling is ‘as if one looks back’ at something (‘having moved’), is in the very process of that mental movement at the very time (‘moving now’), or anticipates it in the future (‘will move there’). This dimension, too, will be easier to grasp soon. Both dimensions can be looked at through the distinction between ‘minimal’ and ‘narrative’ self: sense of vital self-agency or ownership experienced as extended or being devoid of temporal and spatial extension (e.g., Gallagher, 2000). As we shall see, whether a vitality structure can expand to all three (meta)dimensions depends on the nature of the specific structure.

As a final note, I will follow Jaak Panksepp [1998] 2005 and use capital letters for the proposed vitality structures to avoid them being directly associated with the culture, etymology, and meaning of the chosen labels. Although I have tried to find labels that give phenomenological inference about what I am trying to express, it is essential to acknowledge the burdens and limits of language when communicating human experience. In this regard, our topic is not so much different to Panksepp’s attempts to describe affective sensations across animals.

### CLIMB

CLIMB refers to a dynamic feeling of ‘going up’ by means of exerted effort. It overlaps with the general sensation of ‘progress’—which is felt as a *forward*-directed force—yet CLIMB has at least two distinctive features. First, whereas progress may happen automatically by its own (feeling of story progress; moving onwards in space; etc.), CLIMB entails exerted effort that represents an *upward*-directed force. When a player experiences CLIMB, it feels as if “I’m making it” and not merely passively moving to a direction. Common designs that trigger CLIMB in players are the ranked ladders in esports (the ‘ladder’ metaphor is not accidental) and level-up systems in roleplaying games (the post-fix ‘up’ is not accidental).

**Figure 2.**
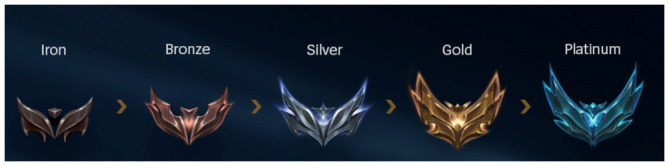
Design that evokes CLIMB. The first half of ranks in LoL. Author’s own screenshot, no further permission needed. © Riot Games.

In LoL and WR, the ranking systems are nearly identical and success is generally referred to as climbing in their player communities (e.g., Kou & Gui, 2014; Siutila & Havaste, 2019). Winning matches accumulates points that eventually lead to higher ranks. Both systems are also visually represented as increasingly *higher* or *taller* ( Figure 2). In these systems, CLIMB is designed and experienced macro-dimensionally: one, two, or three victorious matches do not lead to any change yet, as even the smallest step upward usually requires five or six consecutive wins, i.e. several hours of playtime. More often than not, players cannot win consecutively and reaching the next tier takes several days, weeks, or months. Therefore, experiencing CLIMB via the ranking systems of LoL and WR feels ‘sluggish’, gentle motion in the background until occasionally manifesting from a horizon. CLIMB can be sensed from three different meta-temporal perspectives—retrospectively, prospectively, and in the present—as if “I made it all the way up here” (looking down), “I’m going to be there” (looking up), and “I’m getting there (an immediate feeling when *taking* a step).

CLIMB can be experienced similarly in CR. Currently, the mobile game provides players with two separate ladders, Path of Legends and Trophy Road, which are both presented as ladders to be climbed up. By winning, players earn points that will move their position on the ladder upwards. The former ladder resets season by season (approximately once a month), which makes experiencing CLIMB through it ‘faster’ or more ‘intense’—even a single victory (of a 2–5 minute match) can lead to a step up of one stair. Movement in the latter, in turn, requires several victories as in LoL and WR. As such, experiencing CLIMB in Path of Legends could be described meso-dimensional: a background feeling that is not immediately present yet surfaces right after each match. Notably, the design is structured to often produce CLIMB by a forced visual animation of upward movement as a post-victory cutscene; meanwhile, a post-loss movement downwards is left uncommunicated (to avoid activating negatively experienced vitality structures, perhaps).

In SBs, in turn, CLIMB is primarily designed as a level-up feature: players collect currency that can be spent to level-up their avatar. Here CLIMB is not connected to the player itself but their avatar in control and experienced more distantly in the meta-spatial dimension. As is typical for roleplaying games, level-ups also increase stats, which players can freely distribute; the link between increasing stats and CLIMB together accumulate empowerment (arguably a separate vitality structure; see Linderoth, 2013b). In addition, SBs also allow players to level-up equipment, such as weapons and talismans. Equipment CLIMB, again, feels even further distanced because it is not ‘you’ or your representation as avatar, but rather something you have—an external object. Even though the upward-directed experience is separate from one’s body, the structure of CLIMB remains similar.

In Table 1, I present the phenomenological spectrum of CLIMB through the previously introduced three dimensions. Although more dimensions and their continuous degrees can be added—each vitality has an infinite number of possible manifestations—the present 27 variations of CLIMB should be pragmatically sufficient for illustrating how it can be differently experienced in videogames (and non-videogames).

**Table 1.** Three-dimensional variation of CLIMB. The matrix illustrates 27 versions of CLIMB.

CHRONOTOPE	Close (self)	Distanced (e.g. avatar)	Far (e.g., object)
**Micro** (tiny/instant)	Past/Present/Future	Past/Present/Future	Past/Present/Future
**Meso** (smaller/faster)	Past/Present/Future	Past/Present/Future	Past/Present/Future
**Macro** (large/slow)	Past/Present/Future	Past/Present/Future	Past/Present/Future

### FINAL STRETCH

FINAL STRETCH refers to a specific feeling of a yet-unfulfilled goal at reach, which requires a relatively small effort to be finalised. It is the moment ‘just before’ something is completed or finished, and importantly, one explicitly feels “I could or should complete that” and not only “it’s close” or “it’s getting closer”. One feels a ‘pulling force’ toward closing the gap, as something that is feasible and a ‘sensible’ thing to do (sense referring to both bodily and noematic momentum). A recurring FINAL STRETCH surfaces with CLIMB in level-ups and ranked ladder: as players are about to reach the next level or tier (“just one more win to get there”), they feel a need for ‘completion’ or ‘closure’ that will settle the project momentarily. However, this is just one of the many possible contexts where FINAL STRETCH can occur. For instance, videogames that involve collecting items or progressing a story may likewise trigger FINAL STRETCH (as in ‘cliffhanger’). Unlike CLIMB, the phenomenological structure of FINAL STRETCH does not seem to bend usefully to a past meta-temporal perspective, as the ‘gap’ ceases to exist after it has been closed. Therefore, I only list the present and future meta-temporal dimensions ( Table 2).

**Table 2.** Three-dimensional variation of FINAL STRETCH. The matrix illustrates 18 versions of FINAL STRETCH.

CHRONOTOPE	Close (self)	Distanced (e.g. avatar)	Far (e.g., object)
**Micro** (tiny/instant)	Present/Future	Present/Future	Present/Future
**Meso** (smaller/faster)	Present/Future	Present/Future	Present/Future
**Macro** (large/slow)	Present/Future	Present/Future	Present/Future

**Figure 3.**
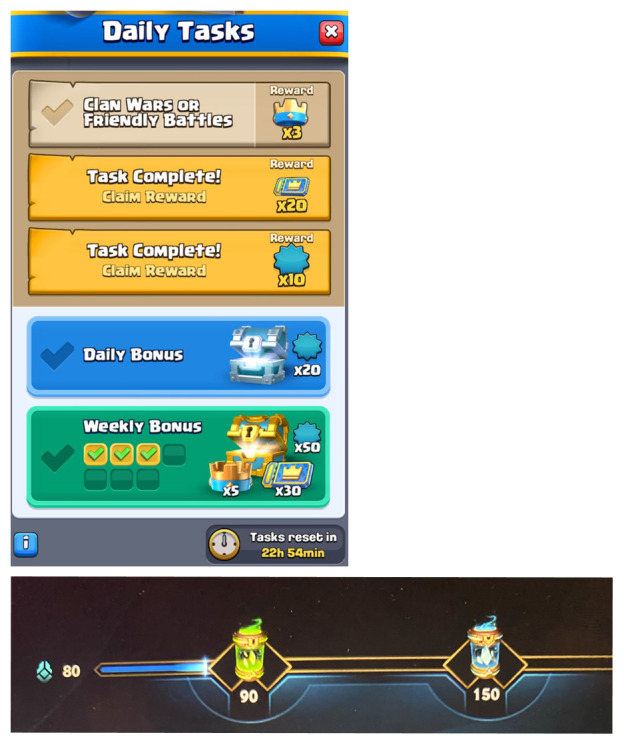
Designs that evoke FINAL STRETCH. After logging into WR, the first daily capsule is almost at reach (below). After logging into CR, the daily bonus is almost at reach (above). Author’s own screenshots, no further permission needed. © Supercell and © Riot Games.

As FINAL STRETCH motivates the player to keep playing, many videogames involve related design structures. In WR, the opening home screen currently rewards players (every day) with two return prizes that accumulate the player’s daily activity points to 80, which is close to earning the green capsule that opens at 90 points ( Figure 3). The feature communicates that reaching the green capsule is near and by playing just one match, the player *will certainly* reach the green capsule. The same reoccurs if the player loses a match: a loss will grant enough points for the green capsule but leaves the blue capsule (at 150 points) out of reach—the player needs to play one more match. I recall many times when my plan was to play one match, but FINAL STRETCH surfaced after a loss and led me to play another match to reach the valuable blue capsule. In all these cases, the FINAL STRETCH is felt near ‘self’ in the meta-spatial dimension.

CR utilises a similar design. The daily rewards on the home screen are based on task activity, and usually two of the three required tasks are possible to accomplish without playing a single match (e.g., visit the shop and open a reward chest). As the third task conventionally requires playing a match ( Figure 3), opening the CR application and collecting directly accessible rewards is clearly designed for FINAL STRETCH, which induces play. This vitality structure, too, operates throughout multiple chronotopic degrees so that smaller and immediate projects are nested inside larger and longer projects: the first reward is only a single click away, the daily reward is only a single match away, and the weekly reward is six days away (and gets closer every day). Moreover, this is but a single form of FINAL STRETCH in CR, as other features such as ranking ladders and the Daily Event promote it too. Currently, players can earn up to 1000 season tokens per day by playing in the Valhalla event; the cap is almost reached by one win, but even five losses are usually not enough to collect all daily available tokens. Experiencing FINAL STRETCH can keep players (like me) playing to reach the cap, which can be simultaneously far and close depending on the win rate.

Immediate and small types of FINAL STRETCH manifest in LoL, WR, and CR. During each match, players control various characters; these characters increase their strength by level-ups, which contribute to their temporary empowerment over time. For example, during a 30-minute match of LoL, the player’s chosen champion can be level-upped 17 times, with several additional upgrades related to equipment. As such, a 30-minute match can involve several instances of FINAL STRETCH when the player feels a need to collect the last bit of experience or gold to reach the next level or equipment update (it can also happen unexpectedly). These ephemeral and instantaneous moments accumulate, until the match ends and all character stats reset—to be reacquired in the next match. By creating such parallel and nested caps, goals, and tiers, designers can incite players re-feel FINAL STRETCH several consecutive times on different experiential dimensions.

Unlike the above, the design structures of SBs rarely provoke FINAL STRETCH. However, there are instances in SBs where FINAL STRETCH can manifest organically. In my own experience, for instance, being close to reaching the next level-up would often trigger FINAL STRETCH and I typically continue playing until reaching that milestone. Arguably, any design that slices progress, rewards, or other anticipation channels into small milestones contributes to FINAL STRETCH by creating the impression that projects are close to completion.

### ALERT

This vitality structure could be named ‘note’ or ‘notification’, but I have chosen to call it ALERT to highlight the active momentum and be more inclusive to various dimensions through which our attention can be alerted to events. Essentially, ALERT is felt as something ‘immediate’ or ‘instant’; it is ‘acute information’ in a sense that one knows it is ‘there now’ and there is a way to access that information. In particular, ALERT refers to that ephemeral and short feeling that rushes through when a player becomes aware of such a signal. Even though conditioning can be part of many ALERT vitality structure forms, it is not simply an activated Pavlovian neutral stimuli paired with a reward, but a felt ‘spike’ or ‘shock’ that has a dynamic form: one feels a need to investigate and reduce uncertainty by instant exploration.

Whereas the structure of CLIMB was previously described as highly flexible in chronotopic, meta-spatial and meta-temporal dimensions—and FINAL STRETCH in chronotopic and meta-spatial dimensions without a past-oriented perspective—the structure of ALERT extends meta-spatially but is fixed to a micro-chronotope without a past-oriented perspective ( Table 3). The phenomenological differences between present and future perspectives can be described continuously from ‘doing it’ to ‘postponing it’; or, a positive ‘surrendering’ response and a negative ‘resisting’ response (see Dalley & Ersche, 2019). In the latter, one feels the dynamic pull that is characteristic to ALERT but counter-pushes or suppresses it in a way that the vital movement slows down or stops. Nonetheless, the nature of ALERT is essentially micro-chronotopic—it is difficult to imagine ALERT being ‘lasting’ or ‘prolonged’ but the feeling is always instant even though it can be postponed to be instantly re-experienced later.

**Table 3.** Two-dimensional variation of ALERT. The matrix illustrates six versions of ALERT.

CHRONOTOPE	Close (self)	Distanced (e.g. avatar)	Far (e.g., object)
**Micro** (tiny/instant)	Present/Future	Present/Future	Present/Future

During play in LoL, levelling up one’s champion periodically triggers ALERT by blinking lights that indicate an immediate opportunity to set new ability points. On the home screen, outside actual play, collectible rewards are communicated with highlighted buttons. These are strongly present especially in the WR mobile version, which also efficiently utilises audio to ALERT players with interactable rapid information, such as friend and match invitations. In these contexts, ALERT serves primarily as a communicative or instrumental function: it informs players of their agency and options for interaction, and very few design features appear to provoke ALERT in intentionally manipulative ways. Only the first daily interactions with the opening home screen, as discussed earlier, involve features that potentially utilise ALERT to encourage returning players to start playing.

**Figure 4.**
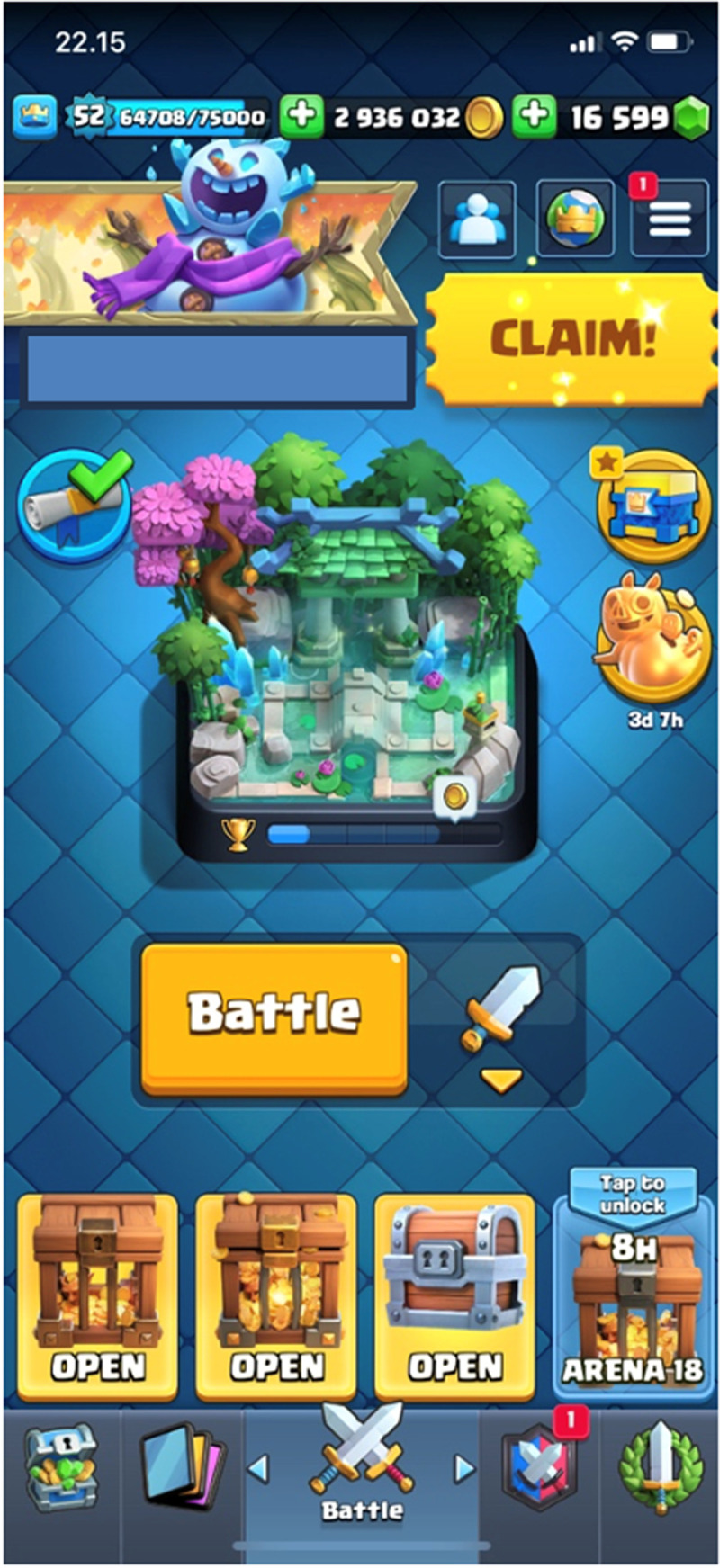
Designs that evoke ALERT. Home screen of CR with a dozen alerting design features. Author’s own screenshot, no further permission needed. © Supercell.

In CR, ALERT has a more central experience function that relates to its design as a mobile game and the expectation that players re-return to play for short periods, several times per day. Currently, my home screen ( Figure 4) shows three ‘shine-animated’ reward chests, one ‘beating’ Claim-button for pending progress rewards, one ‘jumping’ chest animation that implies an unused banner, one ‘pulsating’ encouragement to purchase Hoggy Bank boost for 2,49€, one static red mark that signals a pending friend request, and another static red mark reminding of the possibility to donate cards to a clan member. As this is a very typical number of ALERT-inciting design features for a regular daily log-in, it is clear that ALERT in CR is excessive. This is consistent with the game’s design orchestration: based on its 3-hour and 8-hour chest timers, an active player would open the application every 3–8 hours (at least 3–5 times per day). My subjective rhythm aligns with breaks during work hours and perhaps one or two additional sessions, which adds up to some 20 minutes per day divided into 3–5 sessions. Due to the shortness of these few-minute sessions, tiny ’shocks’ or ‘spikes’ of ALERT easily make a player (like me) click a few extra times per each session; this might not take more than a minute overall but these minutes may easily accumulate into a quarter of total play time and a significant proportion of engagement annually.

In SBs, the design almost completely lacks ALERT features. Only the most essential game-relevant information such as ‘flask upgrades’ are communicated by alerting interface design—even highly important level upgrades have to be manually checked. The difference is consistent with SBs design orchestration: sessions typically last several hours because the progress structure requires lasting engagement. Although it is possible to play SBs in shorter sessions, the challenges are often chained and require time-consuming focus; being frequently alerted would unlikely contribute to the experience but rather distract from it. Compared to LoL, WR, and CR, a core difference is that SBs do not monetise by microtransactions. In the former, ALERT appears to be commonly designed for microtransaction promotion by special offers and new store items, which are merged among other ALERT signals to make players notice sales events. Evidently, the overall design constructions of videogames impact the utility of diverse vitality structures.

## Discussion

My goal has been to propose *vitality structures* as a design-phenomenological framework that can be applied to build construct validity—an in particular, construct clarity and dimensionality—for psychologically meaningful use of technology. I have described three vitality structures (CLIMB, FINAL STRETCH, ALERT), which are part of videogames that have been involved in treatment-seeking. These limited examples demonstrate how different vitality structures can be both related and unrelated to each other, and how they can contribute to gaming experiences. At this point, Linderoth’s (2015) idea of game design as Goffmanian ‘frame orchestration’ is relevant: players frequently switch the ‘frames’ through which they interpret gaming-relevant events, and a core part of design is to orchestrate those frames. As I have shown above and will elaborate on below, there are dissimilarities, for instance, between the orchestration of MOBA, mobile, and single-player roleplaying games, and these variances not only rhythm gaming sessions but also their vitality structures.

**Figure 5.**
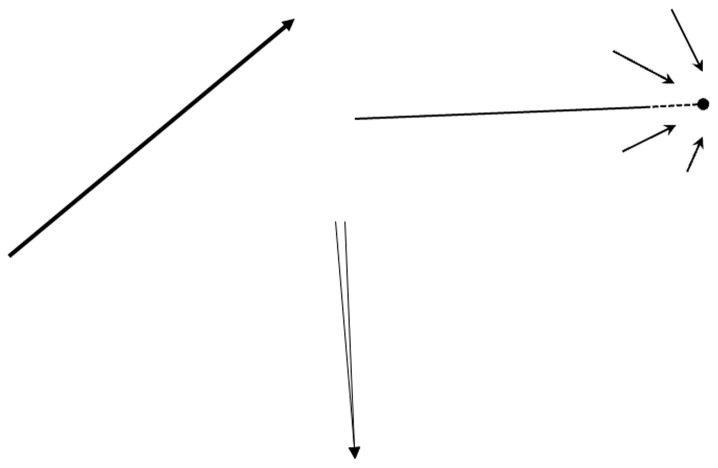
An attempt to visually represent the mental forms of movements in CLIMB (left), FINAL STRETCH (right), and ALERT (bottom). Author’s own graphs, no further permission needed.

The phenomenology of vitality structures is not exclusive to videogames, and can also be experienced in other life contexts. For example, people in academia may feel CLIMB in relation to their careers, FINAL STRETCH when nearing the completion of a study, and ALERT as we receive work-related notifications ( Figure 5). The difference is, videogames and other technological products are explicitly *designed* to generate such vitality, and thus support as well as maintain these experiences—which have proven efficient for controlling or aggregating user engagement. Vitality structures thus apply also to gamification, social media, and other such design contexts, in which their identification can help better understand and experimentally test the effects between selected design-human pairs. Next to such diverse uses in both theory and practice, they also help better model the current clinical discourse: there is no ‘addictive’ substance in vitality structures, but rather designing for certain vitality structures optimises what people experience naturally.

As highlighted earlier, defining and identifying vitality structures is not a matter of truth but an effort of practice. This is not much different from what Cronbach and Meehl (1955) famously said about psychological constructs in general: “The construct is at best adopted, never demonstrated to be *correct*.” (p. 294). There are no right or wrong vitality structures, yet it is possible to collect evidence for the utility of framing vitality structures in certain ways. With that in mind, I do not expect that my present framings of the three chosen vitality structures are final let alone flawless; they are based on how I have subjectively experienced the videogames in question (design ← human) and thus serve as evidence-based hypotheses. Plenty of earlier research have referred to similar phenomena, which contributes to my belief that we are looking at useful directions. I review some of that literature and address the applicability of the design-phenomenological framework.

### Earlier signs of CLIMB

Experiencing CLIMB has been briefly mentioned in various qualitative investigations of gaming-related problems. Already more than a decade ago, Nardi (2010) briefly mentioned level-ups in her ethnographic study on *World of Warcraft* (WoW) in relation to its ‘addictive’ qualities. In another monograph on the same game, Karlsen touches on CLIMB: “Different from gambling, World of Warcraft players are not grinding or questing because of the possible outcome of a random (and lucky) event, but because the outcomes, in the form of resources, act as stepping-stones to other goals” ( 2013). Snodgrass *et al.* (2014) too applied ethnographic methods to study WoW players and some of their accounts reflect CLIMB experiences, such as that of Derek:

*I was playing with my brother because he wanted to get up to as high rank as he could. For an entire summer we put in probably close to 8 to 10 h a day every day. And looking back on that summer, you know, it feels, it feels really kind of hollow. I spent some time with my brother, but it’s almost like a wasted summer, like one of the lost ones I guess in way.* (p. 255)

More recently, Snodgrass *et al.* (2021) also discuss climbing the ranks in *Counter Strike: Global Offensive* in the context of Indian players who self-identified as ‘addicted’ to the game. My own earlier anthropology ( Karhulahti, 2020a) offers in-depth descriptions of CLIMB as ‘anticipation channels’, and in the phenomenological follow-up work with treatment-seekers ( Karhulahti *et al.*, 2022a; Karhulahti *et al.*, 2023b) we found some participants explicitly describing CLIMB as a core source of the problems they had with videogames:

*It’s all about competitiveness [and specifically] climbing the ranks ... even in WoW, which didn’t have the ranking system, it had a third-party Elo system that enabled me to play it competitively [and] get addicted to climbing* (Caius in Karhulahti *et al.*, 2023b, p. 7)

As the examples show, CLIMB-related experiences have been documented in the past, yet without explicitly giving it identity as a construct. The psycho-structural framework by Wood *et al.* (2004) and its update by King *et al.* (2010), for instance, list “leader board features” as one of the numerous “social features” and they define the concept as part of sociality. From another general point of view, thousands of studies since the 1980s have mentioned ‘competition’ as a central part of videogames and their design (e.g., Malone, 1980). Alas, because all videogames can be played ‘competitively’, these accounts have little power to explain competitive design and related phenomenology unless further specified.

As a clinical hypothesis to be developed, I expect that people to whom videogame-related problems concern CLIMB have a significant likelihood to score high on measures of attention deficit hyperactivity disorders (ADHD) and autism spectrum disorder (ASD). A theoretical justification for these links is the established evidence for both disorder categories to phenomenologically involve hyper-level ‘immersion’, ‘fixation’ and/or ‘focus’ for activities that they care about (e.g., Baron-Cohen, 2008; Hallowell & Ratey, 2011). My assumption is that CLIMB—especially with long-term macro-chonotopic forms—is a vitality structure that efficiently connects to these ADHD and ASD human tendencies, both when qualified and non-qualified for a diagnosis.

### Earlier signs of FINAL STRETCH

There appear to be fewer instances that dovetail FINAL STRETCH in the previous literature. Arguably, the structure overlaps with self-efficacy ( Bandura, 1977) where one feels enabled to accomplish a meaningful task at reach. Similar ideas of goal completion have been discussed also in philosophical economics (e.g., Loewenstein, 1999), yet there are few empirical investigations related to it in the research programs on gaming. For example, even though a gambling mechanism like the near-miss (e.g., Pisklak *et al.*, 2020) may look similar—as if ‘almost there’ or ‘almost a win’—it refers explicitly to the past with a certain ‘frustration’ or ‘regret’ afterwards (or accumulation of false excitement). FINAL STRETCH is a toward-pulling feeling that manifests in the present, and can be postponed to the future.

Another similar concept is the relatively much-studied ‘sunk cost’ effect (for a critical overview, see Friedman *et al.*, 2007). The idea of sunk cost is that one’s evaluation of future decision-making becomes biased by the costs (financial, temporal, etc.) invested earlier, e.g. it is more difficult to change a plan after one has already worked on it. There can be conceptual and pragmatic overlap with FINAL STRETCH and sunk cost when a player decides to complete a gaming task (or is inclined to do so); however, for the latter to take place, there should be an alternative ‘lost cost’ scenario that manifests in case of non-completion. For example, in all esports titles discussed earlier (LoL, WR, CR), interrupting a match that has started could logically produce one kind of sunk cost effect, yet this would hardly correspond with FINAL STRETCH. On the other hand, a single-player videogame that allows saving a game state only at predefined locations might simultaneously support both FINAL STRETCH and sunk cost.

In a comprehensive review of clinically relevant design mechanisms, Flayelle *et al.* (2023) taxonomise ‘partial goal fulfilment’ as a class of model-based features. In my interpretation, their model-based design generally refers to technological feedback without randomisation. Design elements that incite FINAL STRETCH could be defined as model-based under partial goal fulfilment. That said, it is important to keep in mind that vitality structures are not design elements *per se* but design-phenomenological constructs that cover a range of possible designs that correspond with an identified vitality. Following Meier and Reinecke (2020), it can be useful to design future research efforts on vitality structures to involve the very interactions carried out by the relevant player population.

My clinical hypothesis is that people to whom videogame-related problems concern FINAL STRETCH have a significant likelihood to score high on measures of obsessive-compulsive disorders (OCD) and impulsivity (see Dalley *et al.*, 2011). Phenomenologically described as a need “to achieve a sense of *completeness*” (OCD by Stein *et al.*, 2019), it would make sense that videogames designed for a ‘sense of incompleteness’—e.g., mobile genres orchestrated by rapid-frequent access rhythm, as discussed earlier—can provoke uncomfortable FINAL STRETCH in people with OCD-related symptoms and tendencies. I see related problems manifest as prolonged gaming sessions by a need to complete ‘one more turn’ or overspending on gaming to receive completion satisfaction at any cost. Although further nosological discussion must be had elsewhere, such instances seem to follow a similar logic as “Obsessive-compulsive or related disorder induced by other specified psychoactive substance” (ICD-11; 6C4E.72) where underlying OCD symptomatology would be activated not by substances but technology use.

### Earlier signs of ALERT

As demonstrated in earlier analysis, in many videogames ALERT appears to serve minor communicative functions. The mechanism of these functions can be partially explained by the predictive processing approach to videogame design ( Deterding *et al.*, 2022), namely, players reducing uncertainty by responding to design features that offer low-cost-high-information value. Although ALERT can be a central part of mobile gaming that operates with high frequencies and short engagement spans, most examples in previous literature seem to come from research on social media applications.

Qualitative studies on social media use have reported various instances where ALERT-like pairs of design and experience manifest. Such instances are often represented as ‘distraction’, yet it is important to stress that ALERT is not negative-valence by default but merely directs attention to a target. Typically, the experience becomes negative when ALERT is unwelcome or felt as excessive due to content or frequency. For example, one of the participants in Mannell’s (2019) interview study, Steve, turns off distracting notifications:

*I don’t have control over when people send me the messages or when I get to see them so I try to do things to control that more. So for example, I try not to log in on Facebook on my app and I turned off all the notification settings for my tablet and my phone.* (p. 82)

Avoiding log-in resonates with my earlier analysis on the potentially overwhelming ALERT in CR. Another frame through which ALERT-like feelings are often discussed is ‘dopamine’ and the related discourse. Being alerted to notifications—whether related to gaming, social media, or other applications—is considered a ‘dopamine boost’, often as a phenomenological metaphor. Conroy *et al.* (2023) interviewed people who experienced their social media use problematic, including Danielle, who describes phone-checking as follows:

*I realized it’s a level of dopamine that gets released when the use of phone (which) brings a sense of achievement ... your dopamine is a very addictive hormone in your body but it may not be for the right reasons* (p. 5)

People like Danielle may thus perceive likes, replies, and other social media notifications brining ‘sense of achievement’ through ‘realease’ but also ‘not for the right reasons’. To what degree this corresponds to ALERT, as I have described it, remains to be explored but the link between ALERT and some of the negative social media experiences seems to be clear. In the study by Vainio *et al.* (2023), people reported various self-limiting strategies for social media use, and these often focused on reducing the effects of features that incite ALERT, such as blinking and bright signal colours that ‘jump’ from the screen when looking at it:

*I had these routines, like putting my phone in another room or if I go to the university, I turn the screen colors off. As I had noticed [problems] in my phone use, I decided not to look at it all during class* (p. 5)

The examples show how researchers have already identified specific design features such as ‘notifications’ contributing to people’s technology use; however, a ‘notification’ is but a single case of design and does not fully represent ALERT as a dual design-phenomenological vitality structure.

My current belief is that ALERT does not play a prevalent role in the experiences of people with clinically significant videogames-related problems. This follows from my axiom that gaming-related problems rarely derive from ‘distraction’ that is associated with ALERT. Nonetheless, as shown earlier, ALERT features can contribute or support larger design structures. I hypothesise that ALERT is experienced as disturbing when associated with high-stress or high-risk content, such as important social interactions. Therefore, it would make sense that negative experiences of ALERT manifest primarily together with tendencies for anxiety, especially in social media use. Following the same logic as earlier, such nosological pattern would be similar to “Anxiety disorder induced by other specified psychoactive substance” (ICD-11; 6C4E.71). In many cases, anxiety being induced by technology use would represent the scenario more accurately than ‘addictive’ behaviour.

### Final remarks: on ontology

Having made it this far, you are likely to have a detailed picture of how vitality structures work, and perhaps an even more detailed list of unanswered questions. In this coda, my goal is to further elaborate on vitality structures and, in particular, what they *are.* Related to this, I will also revisit the methodological challenges regarding their identification.

The problems related to construct development and validation are not new and have been addressed frequently over decades (see especially Chang, 2004; Chang, 2012; Cronbach & Meehl, 1955; Meehl, 1992). More than 70 years ago, Lewin (1951) asserted—with reference psychological constructs, such as ‘frustration’—that

*little attempt has been made to clarify the conceptual properties of those constructs [e.g., if they have] properties of a vector, or a scalar, or a tensor. [We] should be as much concerned with the question of what frustration “is” psychologically, as with the effects of frustration -- psychology, too, can in its own way proceed from the “fire and water” level to a more advanced level of concepts* (p. 23, 35, 37).

Still today, most psychological and psychiatric constructs—including mental conditions and disorders—remain on what Lewin wittily refers to as the fire-and-water-level. Especially clinical research tends to maintain focus on measuring the effects of constructs over the constructs themselves (see Elson *et al.*, 2023; Scheel *et al.*, 2021; Yarkoni, 2022). Although vitality structures do not apply to all psychological domains and are hardly enough to solve the entire problem, they are a step toward better constructs development in the present field. Vitality structures are ‘small constructs’ that function with (and perhaps collectively constitute) bigger constructs; as such, their development serves as a means to start from the smallest solid particles with chronotopically dimensional, comparable properties (Lewin calls a similar set of features ‘conceptual dimensionality’ ^2^).

When explaining the theory of affordances—which shares plenty of conceptual space with vitality structures— Gibson (1977/2014) refers to Wittgenstein and highlights that the duality of affordances “rescues us from the philosophical muddle of assuming fixed classes of objects, each defined by its common features and then given a name” (p. 126). Vitality structures carry the same benefit and allow the paradigm to be recalibrated from the domains of design (elements, patterns, etc.) and experience (factors, typologies, etc.) to *dimensional constructs that bond the two*. As it goes with all progress, the steps are small and the devil is in the details. Both design and phenomenological analyses remain necessary domains of research, but now synergistically bridged by vitality structures.

Affordances are a useful analogy also for deconstructing the ‘particles’ of vitality structures. A chair affords sitting, but only for those who sit and are tall enough. A chair is not an affordance and sitting is not either, but the bonds between specific chairs and specific organisms—when the potential materialises—constitute the very affordance (for a historical review, see Jones, 2003). At this point, Gibson would remind us that it hardly makes sense to define ‘chairs’ in the theory of affordances. This is where the pragmatics of vitality structures diverge from the theory of affordances.

For affordances, there is imbalance between the organism and the environment, as the “organism depends on its environment for its life, but the environment does not depend on the organism for its existence” ( Gibson, 1977/2014, 121). This is obviously incorrect when flowers or other such beings *are* the environment (e.g., for bees). In such interactions, the survival of the environment (flower) depends on the organism (bee) as much as *vice versa*. Both have evolved in time due to the very interaction. ^3^ A similar co-dependency is present in videogames and their designs. Phenomenologists such as Leino (2010) have taken the position even further and demonstrated that many designs very materially *demand* specific interactions from individuals:

*In contrast to certain other technological artefacts which situate themselves in hybrid intentionality relationships, such as pacemakers which are best conceptualised as existing to serve the human, [the] relationship between the game and its player is negotiated primarily on terms dictated by the game artefact. The player, unlike the human carrying a pacemaker, is there to serve the artefact: she can play or not play, but what play implies is often dictated univocally by the game artefact* (p. 244)

Although vitality structures are not limited to those (metaphorically) vital interactions that keep the player-videogame hybrid ‘alive’, it is possible to hypothesise that in some videogames (e.g., esports) a specific vitality structure (e.g., CLIMB) is highly desirable or even necessary for intensive long-term gaming to take place (also consider rotational structures in the original extended mind hypothesis on *Tetris*; Clark & Chalmers, 1998). We might as well distinguish such vitality structures—which serve as a central condition for intensive gaming with a specific videogame or genre—as *prime vitality structures*. It remains for future empirical work to demonstrate how useful that category will be in practice (for a previous attempt to systematically identify similar prime structures, see Järvinen, 2008).

**Figure 6.**
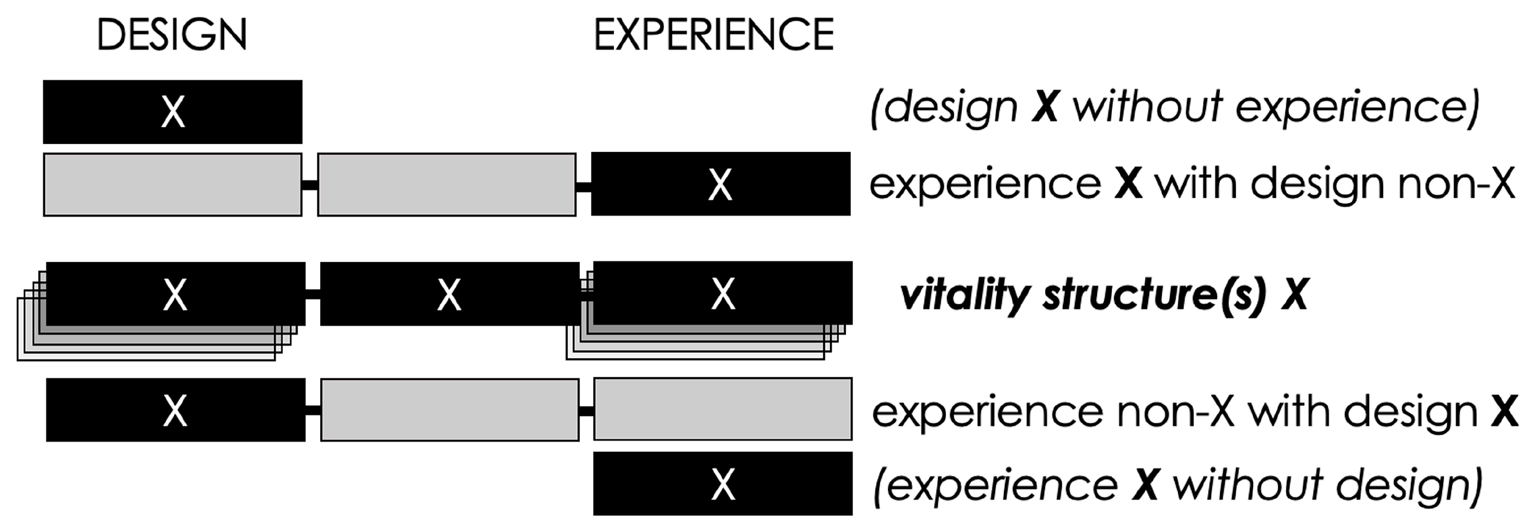
A deconstruction of the vitality structure into three parts (bonded design and experience) and its four incomplete manifestations. Examples using CLIMB: an isolated feature in a videogame designed to evoke CLIMB such as player ranking (topmost), experiencing CLIMB outside videogame play such as in career context (second topmost), player engaged in ranking design but not experiencing CLIMB (second bottom-most), an isolated phenomenological instance of CLIMB. Author’s own graph, no further permission needed.

Following the above, vitality structures materialise when an instance of the identified design meets an instance of the corresponding phenomenological response ( Figure 6). Again, there can be various versions of such design—as I have shown, even ALERT, CLIMB, and FINAL STRETCH have all been designed in different ways both between and withing videogames. Likewise, the corresponding phenomenology constitutes an experiential spectrum within the identified vitality structure. In other words, there is (chronotopic) variation *within* each vitality structure due to the differences in designs and how people experience them; meanwhile, the identified shape of each vitality structure sets limits around both the design and its corresponding phenomenology.

Several recent studies on technology design are empirically consistent with the presented framework by showing how variation in both designs and experiences weave networks of bonds between the two. Vitality structures are identified as selected shapes in such networks ( Figure 7). For example, a large qualitative study on the use of ‘badge’ design in learning platforms found the participants experiencing the same design in nine different ways, concluding that

*game design has multiple potential motivational functions that depend on how users interpret and use them. Therefore, current game design elements like badges are relevant but underspecified: relevant because for many (but not all) users, they do partake in a plethora of motivational functions; underspecified because the mere use of a specific type of game design element alone does not reliably bring forth some (let alone some specific) motivational function in all users* ( van Roy *et al.*, 2019, p. 78)

Other studies demonstrate how background beliefs, cultures, and experiences tend to influence how players experience videogames (e.g., Iacovides & Mekler 2019; Whitby *et al.*, 2023; see Barr, 2008; Karhulahti, 2020b). On the other hand, changes in the design, such as that of difficulty, do not necessarily lead to phenomenological alteration but may be experienced similarly across different variations ( Cutting *et al.*, 2023). For the designer—at least in theory—it might thus be possible to identify a desired vitality structure and connect that to diverse groups of players via designs that are calibrated for individuals respectively (e.g., Bowey *et al.*, 2015; Gerling *et al.*, 2014). This, in turn, reminds us of the other side of the coin: without calibrations, different player groups are likely to end up experiencing one and the same design in multiple different ways.

The above empirical reality leads to a number of logical consequences. First, some vitality structures are more prevalent than others, largely determined by the popularity of the designs that evoke them. Second, vitality structures differ in sensitivity; the potential for any design to evoke a corresponding phenomenology in the target population must range between 0 and 1. Finally, because vitality structures are not natural kinds but pragmatic constructs, researchers and others need to assess the relevance of each new vitality structure—including its prevalence and sensitivity—based on the potential value it can bring to their desired context of practice. The presently addressed clinical discourse and ‘addictive’ design serve as an example of such practice.

In the end, I should briefly poke the last elephant in the room: how does one identify new vitality structures? Having already identified three, the process should be easy to outline, but that is hardly the case. To my knowledge, no simple methods for producing scientific insight exist—the ageless dilemma on falsification continues to be a lack of proper means to identify new theories to-be-subjected to falsification. For the present theoretical work, I made use of my own gaming history and memory to disclose recurring phenomenological entities, which had evolved and sharpened over years of play into three unique vitality structures. Although a philosopher might label some parts of this process as modified phenomenological reduction, I doubt such sources would help many readers interested in embarking on the mission to map out vitality structures. With these caveats, I make two methodological points.

First, the current literature is full of constructs that point toward important design-phenomenological links, albeit not being developed to conceptually capture that duality. Numerous examples have already been mentioned earlier, such as ‘near-miss’, ‘scoring’, and ‘empowerment’, which certainly refer to entities with potential to be refined into testable vitality structures. Elsewhere, for instance, the European Commission (2024) has recently started an investigation into so-called ‘rabbit-hole effects’—likewise a case where the verbal descriptions of the construct capture something relevant about the technology use landscape ( Woolley & Sharif, 2022) but will need more engineering when it comes to conceptual clarity and dimensionality. Although the vitality structure is essentially a dual concept, it cannot be identified without in-depth investigation of the human experience (design ← human), which seems to be currently lacking in the majority of related construct development. Selecting previously discovered ‘proto-constructs’ and investigating their dimensionality with phenomenological player data is a promising means to mine useful vitality structures.

Second, I do not believe that it is feasible to identify vitality structures by most means of currently popular qualitative methods, such as content, thematic, and interpretative phenomenological analysis. As all the above rely on variations of clustering, they can at most direct the researcher to relevant directions. For those interested in utilising empirical datasets, my intuition rather points at abductive approaches, such as that by Timmermans & Tavor (2022) where “the point is to open up moments of surprise and help determine what kind of case you have” (p. 22). Vitality structures are not theories, but they do operate by an iterative hypothesis principle, as being subject to discovery, refinement, and testing. Abductive analysis that explicitly aims to maximise the chance of data surprises should be, in principle, a useful method for such processes (for other previously applied empirical approaches, see Ballou *et al.*, 2022; Petrovskaya & Zendle, 2021; Phillips *et al.*, 2015; Windleharth & Lee, 2020). Ethnographic ( Rapp, 2021) and multiverse-ethnographic ( Karhulahti *et al.*, 2022b) approaches should be possible to optimise for vitality structure identification.

## Conclusions

As a partial solution to the long-term challenges of addressing technology and specifically gaming as ‘addictive’ substances—and as psychologically meaningful interactants in general—I have proposed *vitality structures* as a design-phenomenological framework that can help establish construct validity for related phenomena. Vitality structures are not natural kinds to be discovered but pragmatic, dimensional constructs, which ‘bond’ specific types of design to their phenomenological correspondence. As such, vitality structures serve pragmatic philosophies of science: they are useful as long as they communicate what is both identifiable and empirically prevalent within the target population.

As examples of practice, I have proposed three common vitality structures (CLIMB, FINAL STRETCH, ALERT) that are relevant to videogames that treatment-seeking players have reported as sources of their problems. Each vitality structure was shown to serve distinct functions in relation to differently orchestrated videogames (and non-videogames), thus allowing for specific construct-driven hypotheses on the nature of such designs and their players. The validity of the identified constructs and their links to ‘addicted’ gaming should be investigated separately in clinical studies. I hope the design-phenomenological framework of vitality structures makes related efforts on construct validation and application easier and, perhaps also, possible.

## Ethics and consent statement

Ethical approval and consent were not required.

## Data Availability

No data are associated with this article.

## Footnotes

^1^ To counter the expected critical remarks on what is presented as monodirectional here (design → human or human →design), I feel obligation to add that all relationships like this are bidirectional *de facto* (design →← human). This does not exclude the possibility of paying special attention to one direction.

^2^ I resist temptation to engage with Lewin’s general field theory here and am aware that vitality structures are not immediately compatible with its terminology. However, I am convinced that the principles of field theory will turn out useful in future development of the formalisation and measurement of vitality structures.

^3^ I would not mind classifying literally vital mutualistic interactions, such as those between flowers and bees, as vitality structures of the nonhuman fauna.
