# Supplementary material for: Vitality structures in ‘addictive’ game design
Source: Open Res Eur. 2024 Oct 14;4:47. Originally published 2024 Mar 1. [Version 2] doi: 10.12688/openreseurope.17177.2 (PMC11162526; doi:10.12688/openreseurope.17177.2)
Supplement: Peer review history

# Reviewer report: Reviewer response for version 2

**DOI:** 10.21956/openreseurope.19990.r45138

**Published:** 2024-10-17

**Authors:** Charlotte Eben

**Affiliations:** 1 Department Psychology, Biological Psychology, University of Cologne (Ringgold ID: 14309), Cologne, North Rhine-Westphalia, Germany; 2 Department of Psychology, The University of British Columbia, Vancouver, British Columbia, Canada

Thank you so much for addressing all my comments. For me the article is now much clearer and provides a very useful addition to the field.

 Best wishes,

 Charlotte Eben

Is the study design appropriate and does the work have academic merit?

Yes

Is the work clearly and accurately presented and does it cite the current literature?

Yes

If applicable, is the statistical analysis and its interpretation appropriate?

Not applicable

Are all the source data underlying the results available to ensure full reproducibility?

No source data required

Are the conclusions drawn adequately supported by the results?

Yes

Are sufficient details of methods and analysis provided to allow replication by others?

Yes

Reviewer Expertise:

emotion, gambling, impulsivity

I confirm that I have read this submission and believe that I have an appropriate level of expertise to confirm that it is of an acceptable scientific standard.

# Reviewer report: Reviewer response for version 1

**DOI:** 10.21956/openreseurope.18564.r39666

**Published:** 2024-06-08

**Authors:** Sebastian Deterding

**Affiliations:** 1 Imperial College London, London, England, UK

This work positions itself within the (psychiatric) literature on disordered gaming and attempts to identify the “psycho-structural characteristics” [1] of (gambling) games that afford it. Meanwhile, it speaks to a broader, fragmented effort across fields like empirical aesthetics, health psychology, ecological psychology, HCI, design and architecture, and games research to identify psychologically “active ingredients” in the designed environment. These go under names like collative variables [2], motivational affordances [3], behaviour change techniques [4], game design elements [5], motivational design patterns [6], dark patterns [7], and the like.

 In this work, Karhulahti proposes an analysis of intense game play in terms of certain phenomenological “vitality affects”, identifying CLIMB, FINAL STRETCH, and ALERT as three such qualia that co-occur with particular design features (e.g., CLIMB and ranking systems). This I see as its major contribution: Karhulahti is proposing ideal types of *play experiences* associated with intense gaming and particular design structures as a new starting point or unit of analysis. 

 There is some precedent here: Sundown’s classic “Pilgrim in the Microworld” [8] offers a detailed phenomenological analysis of “the addiction and its thrills” of playing Breakout; Schüll’s [9] cited ethnography of machine gambling delineates being “in the zone” as its core qualia, and related design features like “perfect contingency”; and recent micro-phenomenological studies identify types of “lived experiences of perspective challenging moments” in indie games [10]. Karhulahti cites further ethnographies by Nardi, Karlsen, and Snodgrass.

 Yet none of these texts propose or engage in cataloguing typified engaging experiences across games, and Karhulahti is right that the majority of present attempts in the field start from design features or parameters, not experiences. Hence, I find this project new, exciting, and epistemically worthwhile. It also has practical merit: game designers use experiential vocabularies to understand and guide their practice, like saying that a decision or unit in a board game feels “too crunchy” or “satisfyingly chunky”, or that video game controls feel “tight” or “loose”. Karhulahti’s FINAL STRETCH experience of a goal being almost within grasp and thus pulling us toward some additional play fits right into this list. Reading the provided descriptions, the proposed experiential gestalts and their correlations with certain design features resonate with my own experience, and I haven’t seen CLIMB, FINAL STRETCH, and ALERT documented in this way before.

 That said, the conceptualisation and operationalisation of vitality structures leaves much to be desired, and the present text is not engaging with significant parts of the prior literature – see only the wide range of works on active ingredients in design cited above that the text does not engage with: its references remain in the main contained within gambling research.

 As for conceptualisation, note that my discussion so far focused on vitality affects as experiential gestalts, whereas Karhulahti posits “vitality structures as ‘bonds’ between technology and human experience [...] that bond specific designs with specific experiences” (p. 5). That is because (a) the novelty of his work for me lies in isolating and starting from experiential gestalts or vitality affects, and (b) Karhulahti’s *actual analysis* describes in the main these gestalts, to then identify *several* design features related to them (e.g., ranking systems and level-ups for CLIMB).

 Conceptually, I find vitality structures both intriguing and underdeveloped. I appreciate the dual acknowledgement that (1) relations are always bidirectional (”design →← human”, p. 5) and (2) one can exist without the other: the design feature need not evoke the experience, the experience can be evoked by other circumstances than the design feature (p. 6). 

 But this leaves us to wonder about the intended ontological and causal status and structure and hence, explanatory value of vitality structures. Karhulahti emphasises that they are *not* natural kinds (p. 7, 12). But ontologically, what are they, then? A Weberian ideal type? A real emergent property? Mere labelled regularly observed co-occasions of phenomena with no claimed true causal force or ontological unity (a kind of Humean occasionalism)? If so, the aim of the project would be a humble descriptive one. But Karhulahti seems to want more, both in their stated pragmatist orientation and the express aim “to effectively delineate a design and its underlying clinical mechanism(s)” (p. 6). This at least to me suggests some intent at causal explanation, not just description.

 What then is the proposed causal link between an experience like CLIMB and ranking and level-up systems? The paper points to “affect attunement” to suggest that causality in some respect flows from the person (“design ← human”): “players attune to specific forms of affective engagement that someone designed” (p. 6). This suggests design as a kind of latent potentiality that needs acting on to realise, evident as “an increased probability to trigger a (specific) aesthetic experience” (p. 6). If so, vitality structures feel like an effortful recovery of the concept of affordances as coined in Gibson’s ecological psychology [11] and since widely (re)interpreted and appropriated [12-14]. This conceptual parallel is not explored, which is sad, since the affordance literature arguably has done a lot of the heavy lifting to address the ontological and causal questions Karhulahti leaves unanswered. This is one reason why my own conceptual work and critique of the unquestioned assumption of one-to-one design-experience mappings in gamification draws on affordances [15,16]. (As an aside, there is also an interesting resonance with Kurt Lewin’s field theory as the gestalt structure of lived space from a person’s perspective whose forces and paths determine behaviour [17].)

 Similarly, given that vitality structures assume an attuning active audience, the text misses crucial engagement with empirical and theoretical work supporting that part of what an active audience does is subjectively interpreting/appraising media: there is no discussion of sense-making as a mediator or factor in the design-experience link [18]. This extends to wider discussions in user experience and design research on the holistic, emergent quality of experience [19], to the point that design theorists like Nelson and Stolterman [20] argue that design presents an “ultimate particular” of such high complexity, emergence, and indexical embedding in its context that we cannot rationally isolate and deduce causal elements. Thus, the present paper is a first intriguing pointer at something; a sufficiently developed and contextualised new theory it is not.

 Moving finally to methodology, the paper positions itself as an illustrated theoretical proposal not an empirical study, yet de facto is grounded in some (not reported) phenomenological analysis. Also, it positions its practical aim to offer something “useful” to “establish construct validity for related phenomena” (p. 15), If so, it would have been helpful to read more on how practically relevant subject- and game-spanning invariances or even just resonances in vitality affects/structures would be practically established: what counts as sufficient warrant to pragmatically establish “CLIMB” as a vitality structure? At a minimum, I would have expected for the proposed “design-phenomenological framework” to be positioned within other introspective or “first-person” autoethnographic methods. In fact, it matches what Rapp [21] has described and modelled as an “ethno-designer” who autoethnographically explores games and other digital environments “for identifying effective ‘design elements’, understanding how they work and their ‘experiential effects’”. This context is missing.

 In sum, this is an intriguing idea paper that proposes and illustrates a new analytic focus and unit of engaging experiential gestalts (vitality affects) as part of wider experience-design couplings (vitality structures). It adds to psychological gambling research, and is sufficiently embedded in this body of work. As a potentially multidisciplinary proposal, it lacks crucial engagement with affordances and related literatures in design, HCI, or gamification research.

Is the study design appropriate and does the work have academic merit?

Yes

Is the work clearly and accurately presented and does it cite the current literature?

Partly

If applicable, is the statistical analysis and its interpretation appropriate?

Not applicable

Are all the source data underlying the results available to ensure full reproducibility?

No source data required

Are the conclusions drawn adequately supported by the results?

Partly

Are sufficient details of methods and analysis provided to allow replication by others?

No

Reviewer Expertise:

Game design, games research, motivational psychology

I confirm that I have read this submission and believe that I have an appropriate level of expertise to confirm that it is of an acceptable scientific standard, however I have significant reservations, as outlined above.

## References

[1] : The Role of Structural Characteristics in Problem Video Game Playing: A Review. *Cyberpsychology: Journal of Psychosocial Research on Cyberspace, 4(1), Article 6. https://cyberpsychology.eu/article/view/4229* .2010;

[2] : The Role of Collative Variables in Aesthetic Experiences.2022; 10.1093/oxfordhb/9780198824350.013.20 385-429 10.1093/oxfordhb/9780198824350.013.20

[3] : Technical opinionMotivational affordances. *Communications of the ACM* .2008;51(11) : 10.1145/1400214.1400244 145-147 10.1145/1400214.1400244

[4] : Behaviour change techniques: the development and evaluation of a taxonomic method for reporting and describing behaviour change interventions (a suite of five studies involving consensus methods, randomised controlled trials and analysis of qualitative data). *Health Technol Assess* .2015;19(99) : 10.3310/hta19990 1-188 10.3310/hta19990 26616119 PMC4781650

[5] : From Game Design Elements to Gamefulness: Defining Gamification. Proceedings of the 15th International Academic MindTrek Conference: Envisioning Future Media Environments. *MindTrek 2011. 11. 9-15. 10.1145/2181037.2181040* .2011;

[6] : Irresistible Apps: Motivational design patterns for apps, games, and web-based communities. *Apress* .2014;

[7] : An ontology of dark patterns knowledge: Foundations, definitions, and a pathway for shared knowledge-building. *In Proceedings of the CHI Conference on Human Factors in Computing (pp. 1-22).* .2024;

[8] : Pilgrim in the Microworld. *New York: Warner Books* .1983;

[9] : Addiction by design: Machine gambling in Las Vegas. *Princeton University Press* .2012;

[10] : “Conversations with pigeons”: Capturing Players’ Lived Experience of Perspective Challenging Games. *Proceedings of the ACM on Human-Computer Interaction* .2023;7(CHI PLAY) : 10.1145/3611051 833-855 10.1145/3611051

[11] : The Ecological Approach to Visual Perception.2014; 10.4324/9781315740218 10.4324/9781315740218

[12] : Interactive Media: The Semiotics of Embodied Interaction.2008; 10.1007/978-1-84800-036-0 10.1007/978-1-84800-036-0

[13] : How Shall Affordances be Refined?: Four Perspectives. *Lawrence Erlbaum Associates* .2003;

[14] : Explicating Affordances: A Conceptual Framework for Understanding Affordances in Communication Research. *Journal of Computer-Mediated Communication* .2017;22(1) : 10.1111/jcc4.12180 35-52 10.1111/jcc4.12180

[15] : Eudaimonic Design, or: Six Invitations to Rethink Gamification, in Fuchs, M., Fizek, S., Ruffino, P. & Schrape, N. (eds.), Rethinking Gamification. *Lüneburg: meson press 305-331* .2014;

[16] : Situated motivational affordances of game elements : A conceptual model. *CHI Gamification Workshop 2011. 3-6.* .2011;

[17] : Field Theory in Social Science. Selected Theoretical Papers. Edited by Dorwin Cartwright.1951;

[18] : Collecting Pokémon or receiving rewards? How people functionalise badges in gamified online learning environments in the wild. *International Journal of Human-Computer Studies* .2019;127: 10.1016/j.ijhcs.2018.09.003 62-80 10.1016/j.ijhcs.2018.09.003

[19] : Experience Design: Technology for All the Right Reasons. *Synthesis Lectures on Human-Centered Informatics* .2010;3(1) : 10.2200/S00261ED1V01Y201003HCI008 1-95 10.2200/S00261ED1V01Y201003HCI008

[20] : The Design Way.2012; 10.7551/mitpress/9188.001.0001 10.7551/mitpress/9188.001.0001

[21] : In Search for Design Elements: A New Perspective for Employing Ethnography in Human-Computer Interaction Design Research. *International Journal of Human–Computer Interaction* .2021;37(8) : 10.1080/10447318.2020.1843296 783-802 10.1080/10447318.2020.1843296

# Response

**Published:** 2024-08-29

**Authors:** Veli-Matti Karhulahti

Thank you for all the detailed feedback on various aspects of the article.

 After carefully considering the feedback from all reviewers, numerous revisions and extensions were made that aim to make the work more comprehensive. As a result, the current article is almost 20k words (I think MIT Press books in the Playful Thinking series are around this length). Although such level of depth may keep many readers away, I’m convinced that the added detail will be helpful for the fewer but more devoted readers who are working on the topic, which is what matters in the long run.

 In brief, all sections have been significantly expanded and two completely new sections were written. When finalizing the revisions, I found myself wanting to write more especially about the (so far hypothetical) process of formalizing, operationalizing, and measuring vitality structures in/for empirical contexts, but this would entail yet another new section of thousand(s) words. That is where I drew the line, aware that some questions were still left partially unanswered.

 The work remains a starting point, but a much stronger one from which future progress should now be easier to make.

 Regarding methodology, I have clarified the section with added details about the use of memory. I did not use ethnography or other traditional qualitative methods, albeit I agree they could be useful for a project like this. Based on my experience, a lot of influential work in HCI, games, and related fields involve detailed examples that do not properly disclose their source; in most cases, the source is an author’s memory. I hope the present methodological description can pave the way for more transparent reporting of examples, when they do not derive from systematically planned data and methods. 

 Once again, thank you for all the time with this long article,

 Veli-Matti Karhulahti

# Reviewer report: Reviewer response for version 1

**DOI:** 10.21956/openreseurope.18564.r39616

**Published:** 2024-05-20

**Authors:** Seth Giddings

**Affiliations:** 1 Winchester School of Art, University of Southampton, Southampton, England, UK

This is a rich and clearly argued article that offers significant concepts and methods to research into the intense and multifaceted nature of videogame-player relationships. The author sustains a critical distance from the loaded term 'addiction,' whilst not, as in much game and media studies commentary, dismissing concerns about the deleterious potential of compelling technological experiences as mere 'media effects.' The article's reference to, and building on, the author's earlier empirical work with treatment-seekers, ties the theoretical enquiry to its clinical implications. 

 This reviewer does not have a grounding in the literature and methods of psychiatry, so will take the robustness of the article in this regard on trust. As a game scholar centrally concerned with the intimate interplay or collusion of the human and technological in digital play however, I found much in this article that is thought-provoking and productive. The concept of vitality structures is convincing, with its insistence on game design mechanics and human players not as discreet entities but as generative of contingent but dynamic affective processes. 

 The autographic methods works well, and the author applies and expands the conceptual terms and matrices through vivid and carefully described examples of games and gameplay, and through the three hypothetical vitality structures. Overall this research has much to offer to researchers in the clinical field, in game studies, and in the study of human-technology relations more widely.

Is the study design appropriate and does the work have academic merit?

Yes

Is the work clearly and accurately presented and does it cite the current literature?

Yes

If applicable, is the statistical analysis and its interpretation appropriate?

Not applicable

Are all the source data underlying the results available to ensure full reproducibility?

No source data required

Are the conclusions drawn adequately supported by the results?

Yes

Are sufficient details of methods and analysis provided to allow replication by others?

Yes

Reviewer Expertise:

Game studies, digital media studies, media ethnography, philosophy of technology

I confirm that I have read this submission and believe that I have an appropriate level of expertise to confirm that it is of an acceptable scientific standard.

# Response

**Published:** 2024-08-29

**Authors:** Veli-Matti Karhulahti

Thank you for the feedback from a games research point of view.  I believe it is always valuable to hear from reviewers from different disciplines, as the distribution of content and style tends to read differently depending on one’s background and expertise. Striking an optimal balance in interdisciplinary work is always a challenge. Due to feedback from other reviewers who come from different viewpoints, several changes and additions were introduced to the revised version, which I hope did not move it too much away from the games research readership.  Once again, thank you for all the time with this long article

# Reviewer report: Reviewer response for version 1

**DOI:** 10.21956/openreseurope.18564.r39611

**Published:** 2024-05-09

**Authors:** Max V Birk

**Affiliations:** 1 Eindhoven University of Technology, Eindhoven, The Netherlands

The manuscript outlines limitations in the construct validity when identifying “addictive design.” The author argues that the notion of addictive design hinges on the assumption that identifiable structures in games are psychologically relevant. For example—in my interpretation—we interpret “Loot Boxes” as addictive, but we don’t know if the object, outcome, reward pattern, rewards themselves, the design of the loot boxes, or any combination of these features is psychologically relevant.

 To address and offer a practical approach, the author offers so-called “vitality structures” as a “design-phenomenological framework” to define identifiable and empirically prevalent pragmatic constructs. The author then applies the approach to the three “vitality structures” CLIMB, STRECH GOAL, and ALERT.

 The paper addresses an interesting, difficult-to-grasp, but highly relevant topic—the lack of construct validity in game research, particularly in domains that make broad claims about the effects of game design.

 I find the work to be innovative and considered in its own right. The aim of the work is applaudable, and the interdisciplinary aptitude is visible.

 While I really enjoyed reading the work, there are a few limitations that need to be considered to ensure the scientific quality of the work, the theoretical framing, methodological rigour, and overall accessibility of the work.

1. Background literature. The work is strongly situated in addiction literature and presents state-of-the-art work. However, there are limitations when diving into design and design phenomenology. The phenomenological terminology is used, but the concept is undefined and not situated in the current design or literature on the philosophy of technology, e.g., Peter-Paul Verbeek [Ref 1]or Luciano Floridi [Ref 2], would fit to characterize what “experience” in a technology-mediated world means. Because insights are given into the experience and behaviour resulting from design, empirical aesthetics are relevant
2. for the presented conflict, e.g., (Marcos Nadal et al, 2022) [Ref 3], and the often more empirically focused work conducted in the HCI community, e.g., [Ref 4], [Ref 5], and in game design classification, e.g., [Ref 9]. Overall, I find the embeddedness in the literature leaves room for improvement to do the interdisciplinary claim justice.
3. Flow and readability. The paper is written in a non-scientific, rather personal style, sometimes colloquial style, e.g., “Second, delineating an ‘addictive’ design *per se* as, say, alcohol or nicotine remains a holy grail in the field; also in the sense that we do not know if it exists.” or “This is where I pause and hope you have a tentative picture of what I have in mind when using the term ‘vitality’.” While I find this to be sometimes compelling and interesting, it also distracts me from leaving the argumentation behind and looking at what actually works in the proposed approach and what doesn’t—the personal perspective overshadows the scientific argument.
4. Methods. The methodology of the work does not follow any guidance but is self-constructed. The work is reminiscent of qualitative methods such as grounded theory and thematic analysis but never formally introduces what the approach is. I highly recommend ( Muratovski et al,2022) [Ref 6] to find grounding in design research methods—that provide a bit of context on how to interpret the evaluation of the work, and (Victoria Clarke et al,2013) [Ref 7]. A proper methodological description would very much help to interpret the presented concepts and the author’s reflection.
  1. My main grievance here is with the presented structure (Method, Analysis, Discussion) that makes me believe formal structures are followed, while the authors follow their own flow and presentation style—which works in itself but has the caveat that it is difficult to place what has been done, how, with which results, and to what end.
5. Results. The presentation of the classification itself reveals that there are issues here. For CLIMB the table shows no differences, no pattern, except that PAST/PRESENT/FUTURE are relevant across all dimensions. The approach shows conceptual weaknesses that would have been interesting to carve out and discuss in detail.
6. Vitality forms seem to be an interesting inspiration. However, considering that the dynamic nature of gameplay has been discussed and framed by Hunicke et al [Ref 8], I suggest drawing from frameworks such as Mechanics-Dynamics-Aesthetics to explore the relationship between combined mechanics, i.e., dynamics and aesthetical experiences.
7. Mental health remains the author's application context and self-declared goal, but it remains an application context. From a scientific perspective, the discussion is relevant across domains. I don't suggest reframing the work, but being clear about the utility value would benefit the work.

Minor: FINAL STRECH -> FINAL STRETCH?

 In summary, the setup of the work is very strong, and the synthesis of the work, elements of the discussion and overall insight is a clear contribution to the current discourse. However, the work falls short when looking at the background work, considering the emphasis on interdisciplinarity, and scientific rigour, considering that the ambition of the work is to advance construct validity. Overall, I struggle with the paper as a research article—especially when applying scientific rigour standards to the methods.

Is the study design appropriate and does the work have academic merit?

Partly

Is the work clearly and accurately presented and does it cite the current literature?

Partly

If applicable, is the statistical analysis and its interpretation appropriate?

Not applicable

Are all the source data underlying the results available to ensure full reproducibility?

No source data required

Are the conclusions drawn adequately supported by the results?

Partly

Are sufficient details of methods and analysis provided to allow replication by others?

No

Reviewer Expertise:

I have more than 12 years of experience in game research. I am an HCI research who published on motivation in games, the effect of games on behaviour, and on games in relation to mental health. I have worked on behavioural design in games (https://osf.io/x9vhs/). I have worked in the context of industrial design and game design, and have supervised a range of qualitative projects and published using qualitative methods in relation to design. The philosophical and ontological angle of the work is most distant to me, while I have been exposed to discussions on phenomenological and post-phenomenological design.

I confirm that I have read this submission and believe that I have an appropriate level of expertise to state that I do not consider it to be of an acceptable scientific standard, for reasons outlined above.

## References

[1] : Cyborg intentionality: Rethinking the phenomenology of human–technology relations. *Phenomenology and the Cognitive Sciences* .2008;7(3) : 10.1007/s11097-008-9099-x 387-395 10.1007/s11097-008-9099-x

[2] : The Philosophy of Information. *https://global.oup.com/academic/product/the-philosophy-of-information-9780199232383?cc* .2013;

[3] : The Oxford Handbook of Empirical Aesthetics. *https://global.oup.com/academic/product/the-oxford-handbook-of-empirical-aesthetics-9780198824350?cc* .2022;

[4] : A conceptual framework for understanding and identifying gamblified experiences. *Computers in Human Behavior* .2024;152: 10.1016/j.chb.2023.108087 10.1016/j.chb.2023.108087

[5] : The hidden intricacy of loot box design: A granular description of random monetized reward features.2020; 10.31234/osf.io/xeckb 10.31234/osf.io/xeckb

[6] : Research for designers: A guide to methods and practice. *SAGE Publications* .2022;

[7] : Successful qualitative research: A practical guide for beginners. *https://www.researchgate.net/publication/256089360_Successful_Qualitative_Research_A_Practical_Guide_for_Beginners* .2013;

[8] : MDA: A Formal Approach to Game Design and Game Research. *https://www.researchgate.net/publication/228884866_MDA_A_Formal_Approach_to_Game_Design_and_Game_Research* .2004;

[9] : Pattern Language for Game Design.2020; 10.1201/9781003119029 10.1201/9781003119029

# Response

**Published:** 2024-08-29

**Authors:** Veli-Matti Karhulahti

Thank you for all the detailed feedback on various aspects of the article. I have now revised the work extensively, and the text is now nearly twice as long as the previous version. A significant part of the revisions tackle especially review points 1, 2, and 6, as they go deeper into related concepts, theories, and literature in general. Completely new sections discuss theory and ontology. 

 Regarding point 3, I have kept the first-person tone. I understand it may be unconventional, but considering the length of the work, it is possible that less formal style also makes it more readable. Of course this is a matter of personal preference to some extent. 

 Regarding point 4, I have elaborated on the methods section. It is certainly true that the current journal article format (methods, analysis, discussion) is not optimal for theoretical articles like the present one, which do not use explicitly collected data -- I had to include a methods section for technical reasons. As I did not use any of the traditional qualitative data or analyses, the methods now more openly explain the use of memory, which I believe is among the most frequent but rarely disclosed methods in related fields (HCI, games, etc.) when it comes to providing examples. To my knowledge, this is among the first times such elements are made fully transparent to readership.

 Regarding point 5, I believe there has been a misunderstanding. The matrices were developed to illustrate all possible versions, in which the past-present-future is a dimension with three variations. I have now clarified this.

 Regarding point 7, I have further clarified the applicability of vitality structures and fixed the typo.

 Once again, thank you for all the time with this long article,

 Veli-Matti Karhulahti

# Reviewer report: Reviewer response for version 1

**DOI:** 10.21956/openreseurope.18564.r39614

**Published:** 2024-04-22

**Authors:** Charlotte Eben

**Affiliations:** 1 Department Psychology, Biological Psychology, University of Cologne (Ringgold ID: 14309), Cologne, North Rhine-Westphalia, Germany; 2 Department of Psychology, The University of British Columbia, Vancouver, British Columbia, Canada

The current paper suggests a new research avenue to investigate video game designs that might contribute to addictive behaviors. 

 In my opinion, this is a very neat paper using knowledge from different disciplines in order to further inform research on addictive designs. Importantly, it provides some thoughts on formalization and testable hypotheses that are very much needed in the field of behavioral addictions in general.

 I just have a couple of thoughts that might (or might not) be helpful to further improve this paper:

 - While I like the idea of using design features from gambling as a starting point, to me this section feels a little bit unfinished. Maybe you can elaborate a little more on your choice of this exact illusion of control idea (Illusion of control entails more than just the experience of early wins, see for example Clark L, et. al., 2022 [Ref 1]) and why you chose to only focus on the IoC and post-loss speeding. There are more 'obvious' design features in gambling such as "losses disguised as wins", speed of play in general including the much more prominent post-reinforcement pause etc.

 - I just want to make sure, I understood you correctly: the three vitality structures that you mention and elaborate on, are examples and suggested starting points for how it can look like when using vitality structures to describe and classify video game designs? If that is the case, for me as a reader a short sentence explaining this in your opening paragraph would be helpful. 

 - Do you think this new avenue is facilitating quantitative or qualitative research on video game designs? Or both?

 Minor comment:

 - there is a space missing in the footnote 1: "obligation to add"

Is the study design appropriate and does the work have academic merit?

Yes

Is the work clearly and accurately presented and does it cite the current literature?

Yes

If applicable, is the statistical analysis and its interpretation appropriate?

Not applicable

Are all the source data underlying the results available to ensure full reproducibility?

No source data required

Are the conclusions drawn adequately supported by the results?

Yes

Are sufficient details of methods and analysis provided to allow replication by others?

Yes

Reviewer Expertise:

emotion, gambling, impulsivity

I confirm that I have read this submission and believe that I have an appropriate level of expertise to confirm that it is of an acceptable scientific standard, however I have significant reservations, as outlined above.

## References

[1] : Langer's illusion of control and the cognitive model of disordered gambling. *Addiction* .2022;117(4) : 10.1111/add.15649 1146-1151 10.1111/add.15649 34318962 PMC9292938

# Response

**Published:** 2024-08-29

**Authors:** Veli-Matti Karhulahti

Thank you for all the feedback and especially that regarding the section on gambling. The article has now been revised significantly. The revisions involve an extensive rewriting of the gambling section, which I have reconsidered based the feedback. I have also clarified the role of the three examples and their methodological applicability.  Regarding the minor comment, I was unable to find the typo in my version of the document -- I believe formatting has produced different versions and hopefully the error is not present in the new ones. Once again, thank you for all the time with this long article,

 Veli-Matti Karhulahti
